# Design of a diagnostic system based on molecular markers derived from the ascomycetes pan-genome analysis: The case of *Fusarium* dieback disease

**DOI:** 10.1371/journal.pone.0246079

**Published:** 2021-01-28

**Authors:** Mirna Vázquez-Rosas-Landa, Diana Sánchez-Rangel, Eric E. Hernández-Domínguez, Claudia-Anahí Pérez-Torres, Abel López-Buenfil, Clemente de Jesús García-Ávila, Edgar-David Carrillo-Hernández, Cynthia-Coccet Castañeda-Casasola, Benjamín Rodríguez-Haas, Josué Pérez-Lira, Emanuel Villafán, Alexandro Alonso-Sánchez, Enrique Ibarra-Laclette

**Affiliations:** 1 Red de Estudios Moleculares Avanzados (REMAv), Instituto de Ecología A.C. (INECOL), Xalapa, Veracruz, México; 2 Catedrático CONACYT en el INECOL, Xalapa, Veracruz, México; 3 Colegio de Postgraduados (COLPOS), Montecillo, Mexico; 4 Servicio Nacional de Sanidad, Inocuidad y Calidad Agroalimentaria (SENASICA), Centro Nacional de Referencia Fitosanitaria (CNRF), Tecámac, Estado de México, México; Universita degli Studi di Pisa, ITALY

## Abstract

A key factor to take actions against phytosanitary problems is the accurate and rapid detection of the causal agent. Here, we develop a molecular diagnostics system based on comparative genomics to easily identify fusariosis and specific pathogenic species as the *Fusarium kuroshium*, the symbiont of the ambrosia beetle *Euwallaceae kuroshio* Gomez and Hulcr which is responsible for *Fusarium* dieback disease in San Diego CA, USA. We performed a pan-genome analysis using sixty-three ascomycetes fungi species including phytopathogens and fungi associated with the ambrosia beetles. Pan-genome analysis revealed that 2,631 orthologue genes are only shared by *Fusarium* spp., and on average 3,941 (SD ± 1,418.6) are species-specific genes. These genes were used for PCR primer design and tested on DNA isolated from *i)* different strains of ascomycete species, *ii)* artificially infected avocado stems and *iii)* plant tissue of field-collected samples presumably infected. Our results let us propose a useful set of primers to either identify any species from *Fusarium* genus or, in a specific manner, species such as *F*. *kuroshium*, *F*. *oxysporum*, and *F*. *graminearum*. The results suggest that the molecular strategy employed in this study can be expanded to design primers against different types of pathogens responsible for provoking critical plant diseases.

## Introduction

Phytosanitary problems are considered a primary cause behind economic crop losses around the world [1]. Therefore, accurate identification of the causal agent is a critical factor for implementing actions against a specific disease [2]. The introduction of new species or the eradication of native species within an ecosystem contributes also to phytosanitary problems [3]. One clear example is the accidental introduction to the United States of two species of ambrosia beetles (Coleoptera: Curculionidae: Scolytinae) native to Asia: the polyphagous shot hole borer (PSHB) and the Kuroshio shot hole borer (KSHB) [4]. Both plagues have negatively impacted avocado (*Persea americana* Mill) production [5] and many other arboreal plant species [5–7]. Specifically, the KSHB complex has devastated large areas of riparian forest in the valley of Tijuana [8], and also was first reported in Mexico in 2015 [9] and currently, in this country is considered a quarantine pest.

The ambrosia beetles establish mutualistic relationships with various species of fungi mainly for nutritional purposes. To maintain these symbioses, beetles dig galleries and tunnels into the vascular system (especially the xylem) of host plants, where they farm the fungi to guarantee an available food source throughout their life cycle [10]. In some cases, the symbiotic fungi are the causal agents of some diseases [11, 12]. That is the case of the ambrosia beetles involved in PSHB and KSBH complexes that vectored phytopathogenic fungi of *Fusarium* genus (*F*. *euwallaceae* and *F*. *kuroshium*, respectively) causing Fusarium dieback disease [11, 12]. Since Mexico is the most important producer of avocado [13] and possesses forest arboreal species recognized as potential hosts [7], it is a priority to develop strategies that allow us to prevent, diagnose and eradicate this disease.

Currently, micro- and macro-conidia features, the presence or absence of chlamydospores, colony appearance, and growth rates in different culture media are used to identify *Fusarium* species [14]. In consequence, the diagnosis is time-consuming and requires experts in the field. On the other hand, molecular diagnosis is a rapid, sensitive, specific and efficient alternative [15, 16]; including polymerase chain reaction (PCR) and sequencing techniques. In both cases, a small quantity of DNA is required during the assay. There are several PCR based methods to identify *Fusarium* species [17–22], including methods based on real-time PCR (qPCR) [23] moreover there are some recent approaches based on this technique for the detection of ambrosia fungi species. For example, Carrillo et al. [23] developed a multiplex qPCR assay using hydrolysis probes for the detection of the b-tubulin gene.

A critical step of PCR based methods is the selection of a target gene and the development of specific primers and probes [24]. Most of the targets used for plant pathogenic fungi identification derive from housekeeping genes; which do not reflect sufficient sequence variation for the discrimination between species [25, 26], or derive from genes related to the pathogenesis of the fungi; in that case the specificity of these types of markers depends on the evolutionary history of the gene, which could be common to other pathogens, either by horizontal gene transfer [27], common ancestry, or convergent evolution [28, 29], and therefore non-specific.

Also, techniques based on genotyping have also been employed (i.e., restriction fragment length polymorphism (RFLP) or amplified fragment length polymorphism (AFLP)). These techniques differentiate strains, races, or varieties within a given species, and have been used to generate markers that allow, for example, the identification of groups within the species *F*. *oxysporum* [30]. However, given the anonymity of the markers concerning the genome, numerous strains or isolates are necessary to validate the stability of the markers [31].

Distinct strategies based on comparative genomics are used to identify and design specific targets. Comparative genomics studies revealed a wide diversity of genetic content throughout different biological groups [32–34]. This diversity includes genes shared by all the members of a specific biological group (i.e., species, genus, family), frequently called ‘core’, as well as, genes that are not shared by all the members of the group, which are named ‘accessories’, and are unique genes, present in specific lineages or clades [35]. The term pan-genome encompasses all genes from both groups [36]. These concepts have been applied to find taxa-specific targets in order to develop tools for the control of the pathogens as the vaccines [34]. This method has been used to generate tags for *F*. *circinatum* [37], *F*. *oxysporum* [2, 38], and even strains of *F*. *euwallaceae* sp. Nov. associated with PSHB *Euwallacea sp*. [39]. Despite comparative genomics identifies specific targets, these markers can amplify genome non-coding areas that accumulate variation because they are subjected to less selective pressure. Therefore, this is a potential problem regarding marker specificity.

In this study, we performed a pan-genome analysis of ascomycetes associated with ambrosia beetles and other phytopathogens to find gene markers in order to design primers for the accurate diagnosis of *Fusarium* spp. by PCR. Unlike previous research, we focus on the use of coding regions. We probed the specificity of the primers using three different DNA sources, *i)* a pure culture of different phytopathogenic ascomycetes, *ii)* stems of *Persea americana* cv. Hass artificially infected by *Fusarium kuroshium* and iii) field samples from KSHB infested trees. We demonstrate the specificity of the markers, and therefore, our results bring to light the possibility of using this analysis method to develop an accurate diagnostic system for other types of plant pathogens.

## Materials and methods

### 1. Bioinformatic analyses

#### 1.1. Collection of data

Sixty-three proteomes of phytopathogenic and non-pathogenic ascomycete fungi from public databases were used including the species of interest (*F*. *kuroshium*). Some of them also associated with ambrosia beetles (Table 1). Forty-eight proteomes from the JGI database (S1 Table) [40, 41]; contained gene models without isoforms. For the rest of the species, including the recently reported genome of *F*. *kuroshium* [42], we carried out gene models prediction. Additional information related to strain name isolation environment and their host was also included in S1 Table.

**Table 1:** Species considered for the ascomycete pan-genome analysis.

Name of species	The identifier in the JGI database or GenBank	Number of predicted proteins	Species considered as phytopathogenic	Species related to ambrosia beetles	Reference
*Alternaria alternata*	Altalt1	13086	*		NA
*Alternaria alternata*	Altal1	13469	*		[66]
*Alternaria brassicicola*	Altbr1	10688	*		NA
*Blumeria graminis*	Blugra1	6525	*		[67]
*Blumeria graminis*	Blugr1	6470	*		[68]
*Botryosphaeria dothidea*	Botdo1	14998	*		[69]
*Botrytis cinerea*	Botci1	16447	*		[70]
*Cochliobolus carbonum*	Cocca1	12857	*		[71]
*Cochliobolus heterostrophus*	Cochec5_3	9633	*		[72]
*Cochliobolus lunatus*	Coclu2	12131	*		[71]
*Cochliobolus miyabeanus*	Cocmi1	12007	*		[71]
*Cochliobolus sativus*	Cocsa1	12250	*		[72]
*Cochliobolus victoriae*	Cocvi1	12894	*		[71]
*Cryphonectria parasitica*	Crypa2	11609	*		NA
*Cucurbitaria berberidis*	Cucbe1	12439	*		[73]
*Eremothecium gossypii*	Ashgo1_1	4768	*		[74]
*Eremothecium sinecaudum*	Img_2751185714	4528	*		NA
*Erysiphe necator*	Erynec1	6484	*		[75]
*Fusarium fujikuroi*	Fusfu1	14813	*		[62]
*Fusarium graminearum*	Fusgr1	13321	*		[63]
*Fusarium oxysporum*	Fusox2	20925	*		[64]
*Fusarium pseudograminearum*	Fusps1	12395	*		[76]
*Fusarium redolens*	Fusre1	17051	*		NA
*Fusarium verticillioides*	Fusve2	15869	*		[63, 64]
*Gaeumannomyces graminis*	Gaegr1	14463	*		[77]
*Glomerella acutata*	Gloac1	15777	*		NA
*Hypoxylon sp*.	Hypci4a_1	11712	*		[78]
*Hypoxylon sp*.	Hypco275_1	12256	*		[78]
*Hypoxylon sp*.	Hypec38_3	12534	*		[78]
*Leptosphaeria maculans*	Lepmu1	12469	*		[79]
*Magnaporthe grisea*	Maggr1	11043	*		NA
*Mycosphaerella fijiensis*	Mycfi1	10313	*		NA
*Mycosphaerella graminicola*	Mycgr3	10952	*		NA
*Nectria haematococca*	Necha2	15707	*		[65]
*Ophiostoma novo-ulmi*	Ophnu1	8640	*		[80]
*Ophiostoma piceae*	Ophpc1	8884	*		NA
*Ophiostoma piliferum*	Ophpi1	8564	*		NA
*Protomyces inouyei*	Proin1	7804	*		NA
*Protomyces lactucaedebilis*	Prola1	6726	*		[81]
*Pyrenophora teres*	Pyrtt1	11799	*		[82]
*Pyrenophora tritici-repentis*	Pyrtr1	12169	*		[83]
*Sclerotinia sclerotiorum*	Img_2739368080	14491	*		NA
*Sclerotinia sclerotiorum*	Sclsc1	14503	*		[70]
*Setosphaeria turcica*	Settu3	12028	*		[72]
*Setosphaeria turcica*	Settur3	12547	*		NA
*Taphrina deformans*	Tapde1_1	4609	*		[84]
*Verticillium alfalfae*	Veral1	10221	*		[85]
*Verticillium dahliae*	Verda1	10535	*		[85]
*Esteya vermicola*	Pcdm00000000	7736		*	[86]
*Ceratocystiopsis brevicomis*	Pcdn00000000	5884		*	[86]
*Fragosphaeria purpurea*	Pcdl00000000	8320		*	[86]
*Grosmannia penicillata*	Pcdk00000000	7380		*	[86]
*Raffaelea lauricola*	Pcdg00000000	8869	*	*	[86]
*Ambrosiella xylebori*	Pcdo00000000	5242		*	[86]
*Raffaelea ambrosiae*	Pcdi00000000	9471		*	[86]
*Raffaelea arxii*	Pcdh00000000	9335		*	[86]
*Raffaelea quercivora*	Pcde00000000	7813		*	[86]
*Raffaelea albimanens*	Pcdj00000000	9377		*	[86]
*Raffaelea sulphurea*	Pcdd00000000	7446		*	[86]
*Raffaelea sp*.	Pcdf00000000	9628		*	[86]
*Fusarium kuroshium*	Nhte00000000.2	13777	*	*	[42]
*Graphilbum fragrans*	Llko01000000.1	8628		*	NA
*Leptographium procerum*	Jruc00000000	7787		*	[86]

#### 1.2. Prediction of gene models

The prediction of the gene models followed these steps: first, transcriptomic datasets were assembled in order to generate transcriptional evidence; then, using the AUGUSTUS software, gene models were predicted on the genome sequence by integrating ab initio and evidence-based gene finding approaches [43, 44]. Finally, the optimization/refinement of the gene models were done with the MAKER annotation pipeline [45].

The SRA (Sequence Read Archive) database of the NCBI (National Center for Biotechnology Information) was consulted, and transcriptomic data available for 12 of the 15 species of interest were downloaded (S1 Table). Prior to the assembly, the available transcriptomic sequences of the species of interest were processed by a Python script (https://github.com/Czh3/NGSTools/blob/master/qualityControl.py) to discard low-quality reads. For the study purposes, we use the parameters -q 30 (the minimum quality value allowed from in Phred format), -p 90 (minimum percentage of bases in the sequence of -q quality), and -a 30 (Phred format estimated average quality limit throughout the sequence). When overlapping regions were detected (at least 25 overlapped bases in initial-terminal portions of paired-end reads), we used SeqPrep software (https://github.com/jstjohn/SeqPrep) to obtain a single, more extended sequence. We eliminate orphan reads (those in which one of the paired sequences failed to meet the established parameters), in order to only keep paired sequences. Finally, the sequences were assembled with Trinity software [46].

AUGUSTUS software [43, 44] was used to process the data and generate the first gene models version. Then, the MAKER annotation pipeline [45] was used to improve the gene models. We masked the repetitive DNA regions of the genomes by using RepeatMasker software (http://www.repeatmasker.org). The entries for MAKER included the masked genomes; all of the resulting contigs from the assembly of transcriptomic data, the gene models predicted by AUGUSTUS, as well as a protein database containing complete proteomes of a total of 43 ascomycete fungi (S2 Table). We used the generated gene models in subsequent analyses.

#### 1.3. Prediction of orthologs and pan-genomic analysis

The identification of orthologous and paralogous gene groups (orthogroups) was performed using the GET_HOMOLOGUES [47, 48] pipeline, a software that uses reciprocal/bi-directional BLAST analysis to identify sequences maintaining similarities across different taxa above a certain threshold (in this case, 75% of identity and coverage); after that, proteins with similarities were grouped by using OrthoMCL software [49]. Groups were created using normalized scores based on an algorithm that uses Markov chain models, which allowed for the identification of orthologous and paralogous putative genes. Finally, using the Perl script (parse_pangenome_matrix.pl) included with the pipeline, we identified genes present only in *Fusarium* genera, as well as the specific genes for each of the *Fusarium* species.

#### 1.4. Primer design and *in silico* experiments

We designed primers for diagnosis based on both, species and genera-specific genes. Regarding the primer design for species-specific genes, the proteins tagged as an orphan after the identification of orthologous groups were compared against a database including a total of 1,953,116 ascomycete fungi proteins available from GeneBank using the BLASTp algorithm. All proteins which showed homology with proteins of unknown function (hypothetical or predicted) were discarded from further analyses to avoid bias/errors associated with the computational tools used. Proteins showing an identity equal to or greater than 75% with proteins of other species were also discarded basically because we consider that homologs sequences with an identity greater than this defined threshold, could present regions (motifs) that at nucleotide level have high similarity and as consequence, with a greater probability of not being truly specie-specifics. The remaining proteins were considered as a list of possible targets for primer design. For that purpose, the nucleotide sequences corresponding to predicted gene models were extracted from the genome [complete gene structure, including both coding regions (exons) and non-coding regions (introns)]. Primers were designed using Primer3 software [50, 51]. Optimal size required for primer design was 20 nucleotides (at an alignment temperature close to 60°C); the expected range of the amplicon or expected product was set between 400 and 600 nucleotides. The complete structure of putative target genes was used considering that diagnosis employs mainly DNA as template and an estimate of the PCR amplicon length is required.

For the design of the genera-specific primers, the strategy consisted of analyzing the orthologous groups including only proteins of *Fusarium* species. First, the candidate proteins were compared against the ascomycete fungi protein database available on GeneBank and homologs identified as hypothetical or predicted proteins were discarded from further analyses. After this, using Kalign [52], we aligned the proteins contained into the remaining orthogroups and only in those cases that contained proteins, represented the proteins from all available species of the genus of interest. The protein sequences were translated into nucleotide sequences using the backtranseq application included in the EMBOSS [53] software package, to finally be realigned using ClustalW [54] based on specific codon usage. Gblocks [55] was utilized to extract highly conserved regions based on this alignment, and a consensus sequence was generated using the em_cons application included in EMBOSS [53]. Genera-specific primers were designed in these consensus regions using Primer3 [50]; optimal primer length was 20 nucleotides, and the expected product-length range was set between 200 and 1,000 nucleotides.

Finally, we performed an *in-silico* experiment to test specificity. Briefly, the designed primers were tested using ThermonucleotideBLAST [56], which performs a DNA fragment search in a database; however, as opposed to the BLAST [57] algorithm, ThermonucleotideBLAST uses additional alignment parameters based on biochemical variables present in PCR reactions, especially free energy and alignment temperature. A database containing all ascomycete genomes available in GenBank was used as a source of target sequences to perform *in-silico* amplification. We selected primers lacking mismatches regarding the species or genera we were expecting for as well as those whose estimated alignment temperatures were similar for both forward and reverse primers.

### 2. Experimental validation

#### 2.1. Biological material

Different phytopathogenic *Fusarium* strains were used for the experimental validation of the designed primers. The *Fusarium verticillioides* (MY3) strain was provided by Dr Javier Plasencia, School of Chemistry, National Autonomous University of Mexico (UNAM) [58]. The *Fusarium oxysporum* (CB-36) strain was provided by Dr Gloria Carrión, Biocontrol Laboratory, Institute of Ecology A.C. (INECOL). *Fusarium kuroshium* (strain HFEW-16IV-019) was provided by the Mycology Department of the National Reference Center (CNRF). The *Fusarium sp*. associated with *Xylosandrus morigerus* INECOL-BM-04 strain and other phytopathogenic fungi such as *Botrytis cinerea* and *Neofusicoccum parvum* (isolated from *Liquidambar styraciflua*), *Fusarium tricinctum* and *Alternaria alternata* (isolated from *Persea schiedeana* and *Nectandra salicifolia*, respectively) were provide by Dr. Diana Sánchez-Rangel, Phytopathology Laboratory of the Molecular Studies Network (REMAv) at INECOL. All designed primers were tested using DNA isolated from the different fungal species mentioned above as a template; these fungi were grown under controlled conditions, using papa dextrose agar (PDA) medium.

With the purpose of validating the primers designed as a diagnostic tool of Fusarium Diseases (FD), first, the primers were probed using DNA isolated from artificially infected avocado stalks (*Persea americana* cv Hass) with *F*. *kuroshium* HFEW-16-IV-019 strain. Briefly, stalks of approximately 30–40 cm length and 1 cm diameter were collected from one-year-old healthy avocado trees which were grown in pots and then acclimated for 4 to 6 months in a greenhouse. In order to mimic the ambrosia beetle, the stalks were cut into fragments of 3.5–4 cm length and were drilled into the center with a 1/16” Dremel^®^. Then, the stalk-segments were placed into humid chambers and were inoculated into the drilled injury with 40 μL of conidial suspension (1x10^8^ conidia/mL). After 14 days (at 27°C and 16 h light/8 h dark), DNA isolation was carried out.

In addition, tissue from some branches collected from visibly symptomatic trees in the field was used. In Mexico and USA, the highest incidence of affected trees by KSHB corresponds to species such as coral trees and dwarf coral trees (*Erythrina corallodendron* and *Erythrina humeana*), California sycamore (*Platanus racemosa*), coast live oak tree (*Quercus agrifolia*), and some different species of willow trees (*Salix* spp.). All these species are recognized as suitable reproductive host trees of KSHB and susceptible to *Fusarium* dieback (https://ucanr.edu/sites/eskalenlab/?file=index.html). Therefore, with the help of Mexican phytosanitary authorities, was performed a visual examination in urban landscapes and natural, agricultural, and riparian areas in order to identify some trees of the species mentioned above with symptoms of the *Fusarium* dieback disease and/or visible damage caused by KSBH complex. Interestingly, after our search, only symptomatic/damaged trees from *Erythrina corallodendron* were found in one of the evaluated areas (S3 Table). Collected samples were moved and processed at the CNRF under biosafety conditions.

#### 2.2. DNA extraction

Fungal spores were preserved at -80°C in 10% glycerol. These were inoculated in PDA medium (potato, dextrose, agar) to promote germination and mycelial growth. After 15 days, a 0.7 cm^2^ plug from the outer zone of the colony was punched with a sterile well cutter and transferred to new PDA culture plates and incubated for one week in total darkness at 28°C using a CB 210 CO_2_ incubator (BINDER^™^). Mycelium was peeled off from the agar surface with a scalpel and DNA was extracted using a previously reported method [59]. DNA concentration was calculated using a NanoDrop 2000 (Thermo Scientific) spectrometer.

To obtain DNA from infected avocado stalks, the biological material was frozen in liquid nitrogen and then pulverized using a mortar and pestle. Approximately 10 mg of pulverized tissue was employed for DNA extraction using a Plant/Fungi DNA Isolation Kit (Norgene Biotek Corporation) following the manufacturer’s instructions. Obtained DNA was resuspended in 50 μl of deionized sterile water, and its quality was quantified and evaluated using a NanoDrop 2000 (Thermo Scientific) spectrometer. Finally, DNA was stored at -20°C.

In the case of field samples, tree branch segments were cut and lengthwise scraped to extract the inner part of the galleries drilled by the beetles and colonized by the fungi. DNA extraction was performed following a standard protocol [60]. Briefly, 200 mg of plant material was mixed with 1 mL of lysis buffer (Tris-HCl 100 mM pH 8.0, 3 M NaCl, 3% CTAB (cetyltrimethylammonium bromide), 20 mM EDTA and 50–80 mg of polyvinylpyrrolidone (PVP)). Following, a total of 500 μL of chloroform: isoamyl alcohol (24:1) was added and mixed by inversion followed by centrifuging 10 min at 3,500 rpm. The supernatant was mixed with one volume of isopropanol, incubated for 10 min at -20°C. Subsequently, the isolated genetic material was visualized in a 1.5% agarose gel electrophoresis. The molecular weight estimation was carried out using a 1 kb molecular weight marker (Thermo Scientific).

#### 2.3. PCR amplification of ITS region and diagnostic markers

DNA quality was confirmed amplifying the internal transcribed spacer (ITS) region, using the primers ITS1 (5’-TCCGTAGGTGAACCTGCGG-3’) and ITS4 (5’-TCCTCCGCTTATTGATATGC-3’) [61]. The amplification was performed with 10x-Mg PCR buffer, 10 μM of each primer, 50 mM of MgCl_2_, 10 mM of deoxyribonucleotides mix (Sigma), 50 ng of DNA, 2 U of Platinum^™^ Taq DNA Polymerase (Invitrogen) and sterile deionized water for a final total volume of 25 μl. PCR reactions were carried out in a thermal cycler (Applied Biosystems 9700) as follows: an initial denaturing step of 95°C for 3 min., followed by 35 cycles of continual changes beginning with denaturing for 35 seconds at 94°C followed by an alignment step of 45 seconds at 58°C and finally, elongated for 1 minute at 72°C, and an additional elongation step of 5 min. at 72°C after the 35 cycles. The amplicon was analyzed in an agarose gel at 1.5% and the PCR product was sequenced at the CNRF Molecular Biology laboratory.

Likewise, to validate the marker’s design, PCR reactions were performed with 10x-Mg PCR buffer, 10 μM of each designed primer per marker, 50 mM of MgCl_2_, 10 mM of deoxyribonucleotide mix (Sigma), 100 ng of DNA, 2 U of Platinum^™^ Taq DNA Polymerase (Invitrogen) and sterile deionized water for a final total volume of 50 μl. A thermal cycler (Eppendorf Mastercycler Nexus gradient) was used to carry out PCR reactions using the following thermal profile: an initial denaturation of 94°C for 2 min., followed by 35 cycles of continual changes beginning with a denaturation step of 35 seconds at 94°C followed by an alignment step of 35 seconds at 55°C, and an extension step of 1 minute at 72°C and an additional extension step of 5 min at 72°C after the 35 cycles. Finally, the amplicon was analyzed in agarose gel of 1.5% and it was visualized in a Gel Doc^™^ EZ imaging tool (BIO-RAD) and images were processed using Image Lab^™^ (BIO-RAD).

## Results and discussions

### 1. A diagnosis system based on comparative genomics

#### 1.1. *Fusarium* complex shows the largest number of coding genes among ascomycetes

The number of coding genes (CDS) inferred for the ascomycete species analyzed ranges from 4,528 CDS in the case of *Eremothecium sinecaudum* to 20,925 in the case of *F*. *oxysporum*. Consistently with previously reported, 13,777 CDS were predicted into the *F*. *kuroshium* genome [42]. The predicted number of genes for *F*. *kuroshium* resulted comparable to genes reported for *Nectria haematococca* (also referred to by its asexual name *F*. *solani*), *F*. *verticillioides*, *F*. *fujikuroi*, *F*. *graminearum*, and *F*. *pseudograminearum* (15,707, 15,869, 14,813, 13,321, and 12,395, respectively) and slightly lower than *F*. *redolens* and *F*. *oxysporum* (17,051 and 20,925, respectively) (Table 1). Interestingly, the species belonging to *Fusarium* genus are those with the highest number of protein-coding genes and from the total of compared species on this study, their genome sizes are ranged between 36.44 Mb and 61.35 Mb [62–65]. Certain tendencies can be observed between genome size and genes content, a relevant aspect when the core genome and accessory or variable genomes of the species from *Fusarium* genus are defined.

It is also remarkable that the number of gene models predicted according to our analysis, was in the same range as previously reported by Vanderpool *et al*. [86]; especially for the species of *Esteya*, *Ceratocystiopsis*, *Fragosphaeria*, *Grosmannia*, *Ambrosiella*, and *Raffaelea* genus (Table 2).

**Table 2:** Comparison of the number of gene models predicted in the present study and previous reports.

Name of species	Gene models identified in the present study using the described method.	Gene models identified by Vanderpool *et al*. [86]
*Ambrosiella xylebori*	5,242	6,503
*Ceratocystiopsis brevicomis*	5,884	6,327
*Esteya vermicola*	7,736	8,012
*Fragosphaeria purpurea*	8,320	8,493
*Grosmannia penicillata*	7,380	7,284
*Raffaelea albimanens*	9,377	9,715
*Raffaelea ambrosiae*	9,471	9,913
*Raffaelea arxii*	9,335	10,816
*Raffaelea lauricola*	8,869	9,553
*Raffaelea quercivora*	7,813	8,003
*Raffaelea aguacate*	9,628	10,194
*Raffaelea sulphurea*	7,446	7,774

#### 1.2. The ascomycetes pan-genome analysis reveals target genes useful for the identification of *Fusarium* spp.

**Fig 1:**
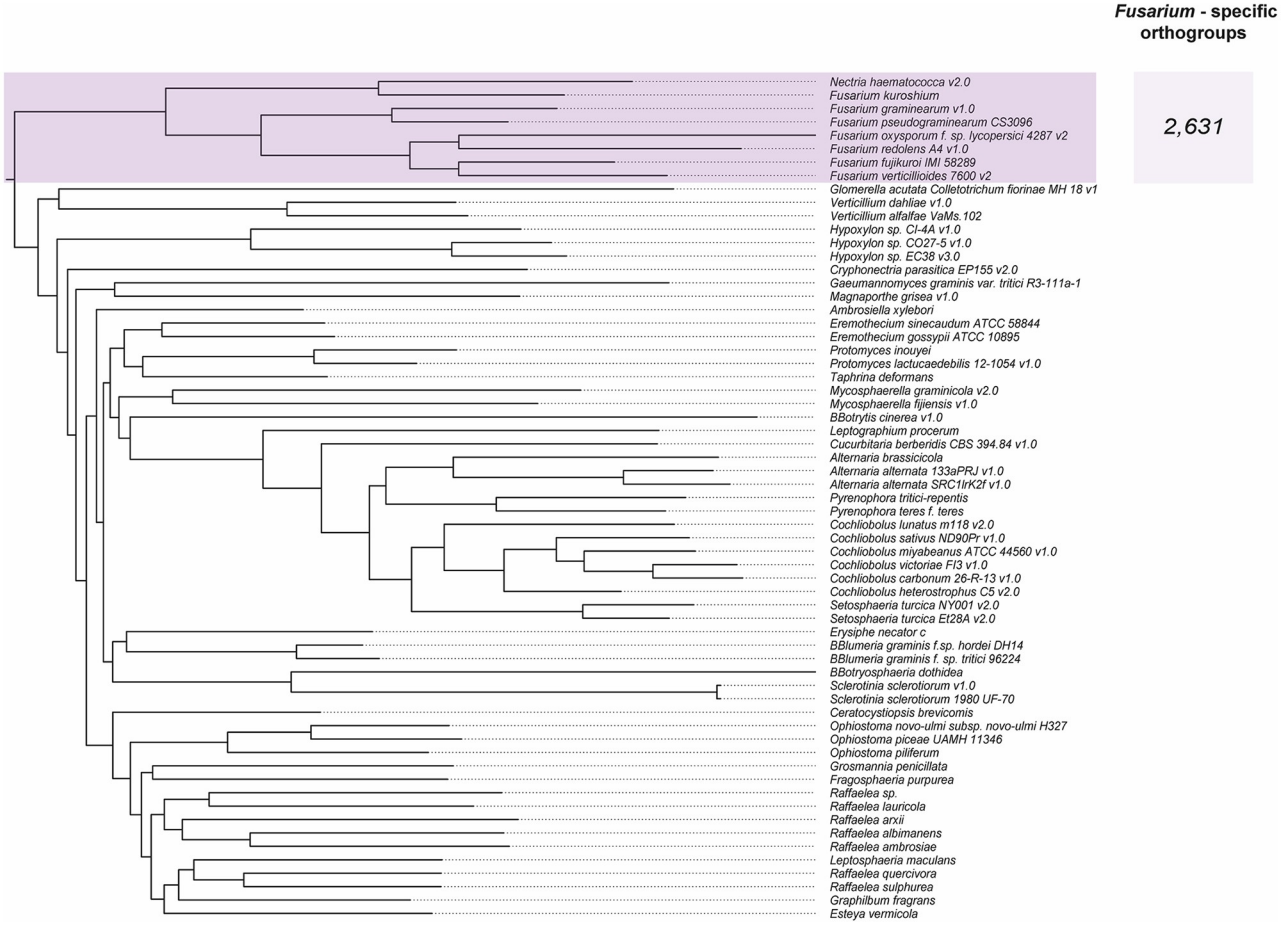
Clustering analysis of ascomycete pan-genome. Clustering based on gene content of the orthogroups inferred. The *Fusarium* complex is highlighted in purple among the number of exclusive genes.

**Fig 2:**
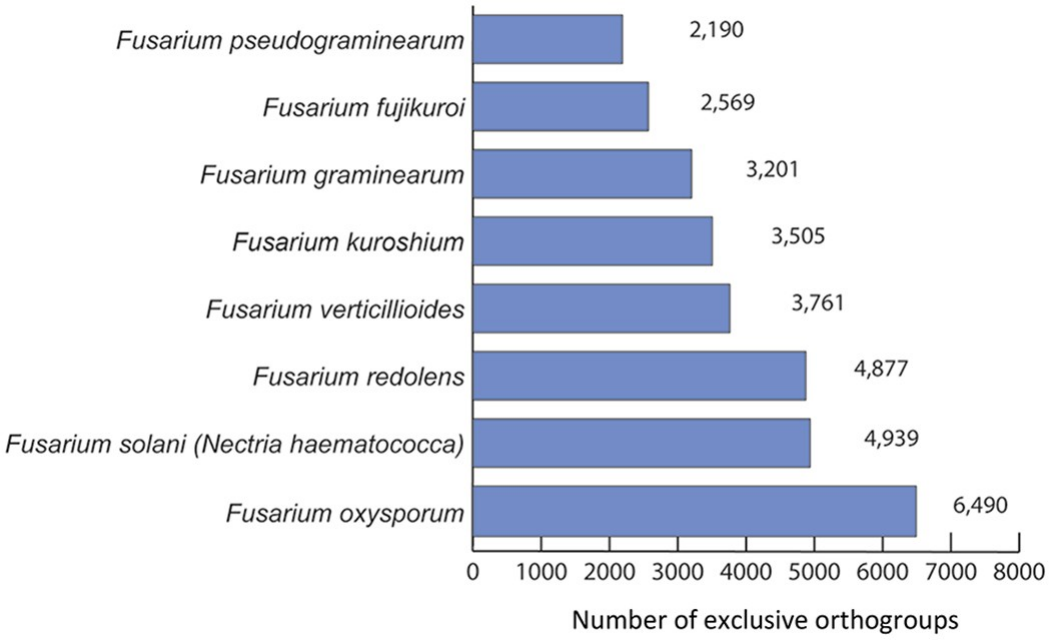
Number of exclusive specie-specific orthologue groups for each member of the *Fusarium* complex. Bar plot including the number of exclusive orthogroups for each *Fusarium* spp.

For the sixty-three proteomes analyzed (Table 1), we obtained a total of 685,096 proteins classified into 382,502 orthogroups or OrthoMCL-defined protein families. Clustering analysis of pan-genome showed that under the set parameters (see Methods section for more information), 2,631 orthogroups, contain unique proteins only from species of *Fusarium* genus (Fig 1). Regarding species-specific proteins, on average 3,941 orthogroups resulted like specific for each of the species included within the *Fusarium* complex (Fig 2). As expected, the number of orthogroups with shared proteins between some of the compared species is smaller, to the extent that the number of species included in the analysis is increased.

**Fig 3:**
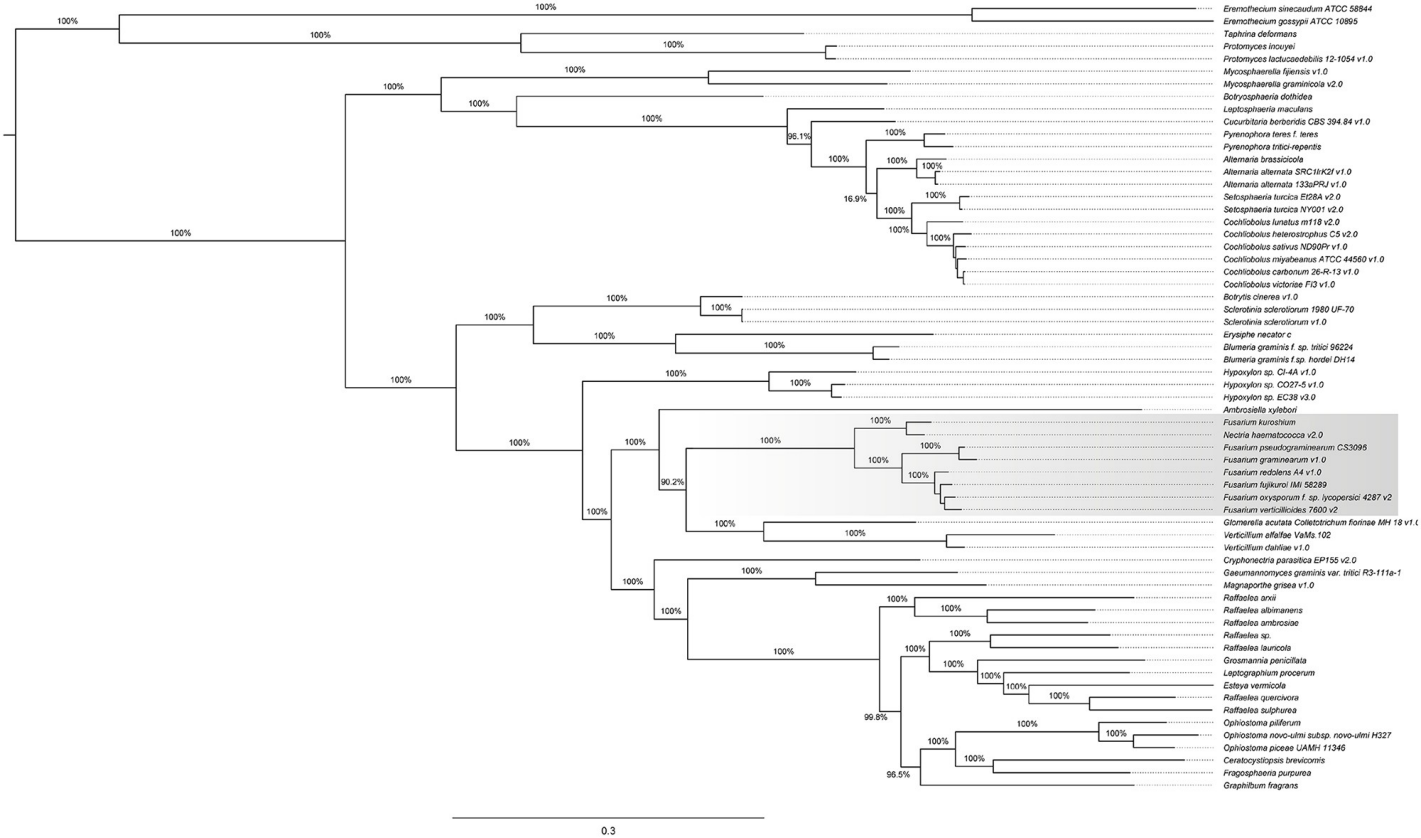
Core phylogeny of ascomycetes. Phylogenetic reconstruction from the 65 core orthogroups.

Moreover, we identified a total of 65 proteins (or genes) belonging to ascomycetes core genome (considering all species used in this study). Core genes fulfill two essential conditions; all the species share them, and they are single-copy ortholog genes. We observe that the topology of the resolved phylogenetic tree (Fig 3) resembles in some clades (but not all of them) to the clustering based on the gene content of the orthogroups inferred (Fig 2). We explain this discrepancy by the differences in the analysis; on the one hand, the phylogeny of core genes shows the evolutionary relationships of species, while clustering analysis of pan-genome genes shows the similarities regarding the presence and absence of shared ortholog genes, which are inferred based on specific parameters.

#### 1.3. *In-silico* validation of a set of primers design for the diagnosis of *Fusarium* spp.

We performed primers design for both, specific species, and *Fusarium* genus. In the case of the species-specific dataset, we identified on average 136 (SD ± 108) target genes per species for which we performed the design of the primers. However, for each species on average, only 120 pairs of primers met the set criteria, i.e., lacked mismatches and the amplification temperatures required by both forward and reverse primers were in the same or equivalent range. Subsequently, based on a manual evaluation considering the alignment temperatures, percentages of guanine and cytosine, and a total lack of secondary structures formation such as hairpins, dimers, cross dimers or palindromes, we selected the primer sets for the experimental tests presented in Table 3.

**Table 3:** Primers designed to be used as specific gene markers in the diagnostic system.

	ID	Description	Forward primer (Fwd)	Reverse primer (Rev)	Expected length (bp)	Mismatches		T_m_		Species
						Fwd	Rev	Fwd	Rev	
**Genus-specific primers**	FuSp01	ATP-dependent RNA helicase MAK5	AACgAYgAggAgAAgATggC	ATAAAgTgCTCCCTCTTDgC	620	1	2	59.83	57.57	*Fusarium spp*.
	FuSp02	transcriptional activator SPT7	CATCgATgAggACgACTACg	CCTCCTggTTCACgTTCTCC	590	2	1	57.7	57.48	
	FuSp03	phosphoribosylaminoimidazole-succinocarboxamide synthase	ACAAgggCAAgATCTTgACC	TCCTTggTgAgCCAgTTgC	750	1	2	57.79	59.24	
	PC013	Positive Control 01, Fa+7/Ra+6	AACgTCgTCgTCATCggCCACgTCgACTCT	ACATACCAATgACggTgACATAgTAgCg	570–600	Karlsson *et al*., 2016 [87]				
**Species-specific primers**	FuKu01	Metal-dependent amidase	ggACAAATgCCTTggATgCg	CCTTTgCCACATTggATCgC	600	0	0	60.13	60.13	*F*. *kuroshium*
	FuKu02	Putative beta-glucosidase M	CCgTCCgAgTAgTCATCTgC	ATgTCAACAgCACCCTACCg	599	0	0	59.83	59.94	
	FuKu03	Amino acid transporter	AAgACgAgTgCAgAgATggC	CgCCTTCTgTTgCTgTATgC	466	0	2	46.52	40.67	
	FuGr01	2-isopropylmalate synthase	CAAACTgCgggTgTgTTAgC	gACTCCAggTCgCTTTCTCC	549	0	0	59.87	59.93	*F*. *graminearum*
	FuGr02	Voltage-gated potassium channel subunit beta-1	gCTACAgCCTggAggTATgC	TATggCgTCggTCgATTTCC	509	0	0	60.2	60.22	
	PC02	Positive Control 02, GOF/GOR	ACCTCTgTTgTTCTTCCAgACgg	CTggTCAgTATTAACCgTgTgTg	435	de Biazio *et al*., 2018 [88]				
	FuOx01	ULK protein kinase	AgACCTCgCTggACAATTgg	ATggTgTTgTgCCCCTTAgg	554	0	0	59.94	59.94	*F*. *oxysporum*
	FuOx02	threonine aldolase	ACgTCTgCCCTATCAACTgC	ACTgTggCTTgAgAgATggC	556	0	0	60.01	59.91	
	PC03	Positive Control 03, 172F/447R	gATCTCTTggCTCTggCATC	CTCTCCAgTTgCgAggTgTT	280	Mishra *et al*., 2013 [89]				

### 2. Experimental validation of the diagnosis system

In order to probe the specificity of the primers designed, we employed DNA from three different sources: *i)* DNA from different ascomycetes fungi strains which were isolated from a pure culture, *ii)* DNA isolated from *Persea americana* cv Hass stalks artificially infected with *F*. *kuroshium* and *iii)* DNA isolated from plant tissue collected on the field from trees apparently infested by KSHB that showed FD symptoms.

#### 2.1. The diagnosis system accurately identifies *Fusarium* spp.

**Fig 4:**
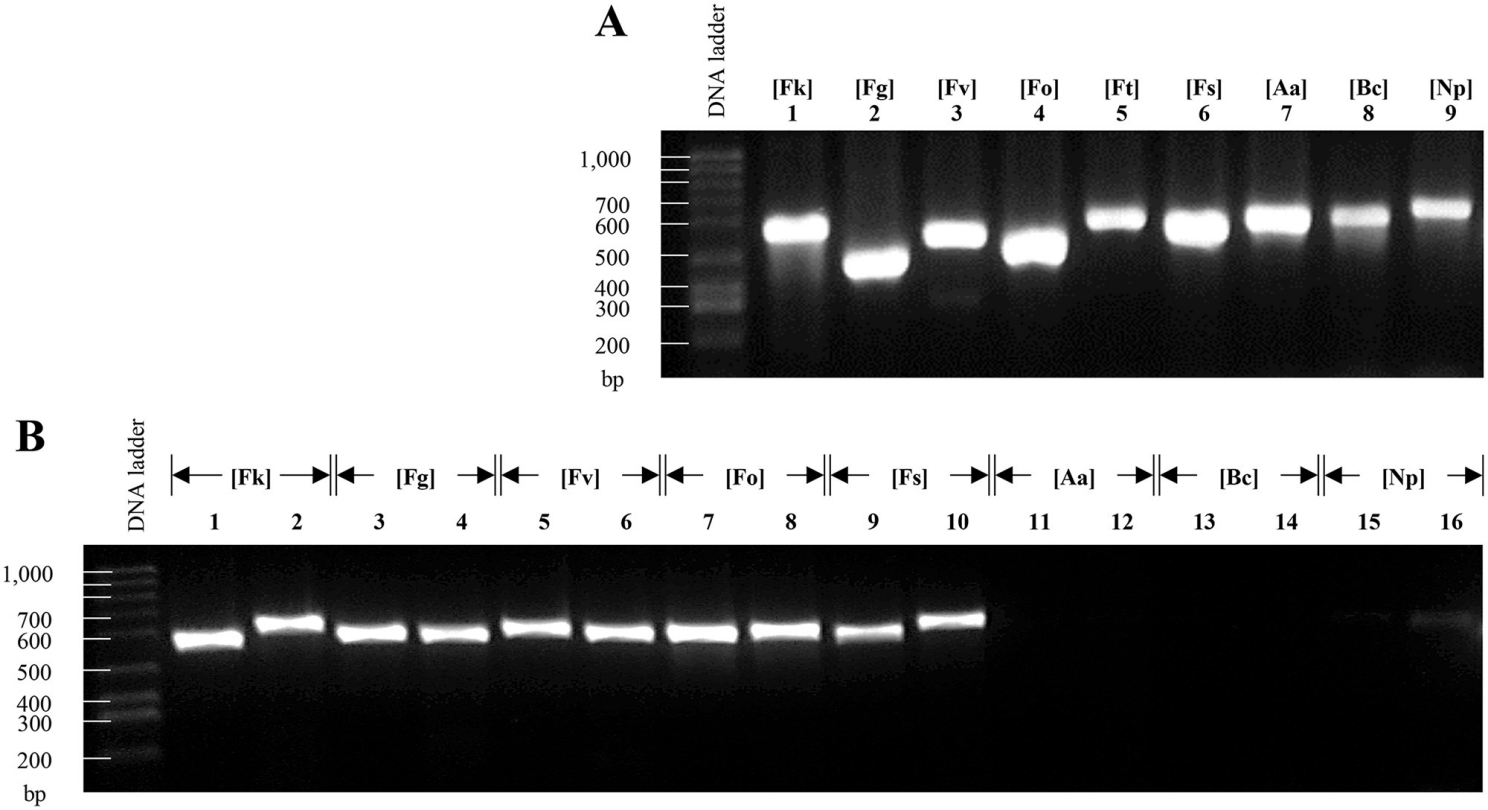
PCR-based assays using FuSp02 and PC01 [87] as diagnostic markers of fusariosis. Agarose (0.8%) gels electrophoresis which shows: **(A)** The amplified ITS region to validate the quality of the template DNA used and isolated from different species of ascomycetes fungi (*F*. *kuroshium* [Fk], *F*. *graminearum* [Fg], *F*. *verticillioides* [Fv], *F*. *oxysporum* [Fo], *F*. *tricinctum* [Ft], *F*. *solani* [Fs], *A*. *alternata* [Aa], *B*. *cinereal* [Bc], and *Neofusicoccum parvum* [Np]; lines 1–9, respectively), and **(B)** The PCR fragments (amplicons) obtained for FuSp02 (odd lanes) and PC01 (even lanes) markers, both of them highly-specific to identify species from *Fusarium* genus. In **(B)**, lanes from *F*. *tricinctum* were not shown only for lack of space reasons in the electrophoresis gel.

Based on the criteria described before (see Methods for details) and after their *in-silico* validation, we selected three primer pairs as the main candidates to identify species of the *Fusarium* genus. These markers were tagged as FuSp01, FuSp02, and FuSp03 (Table 3). We tested the specificity of those primers with DNA obtained from strains of *F*. *kuroshium*, *F*. *graminearum*, *F*. *verticillioides*, *F*. *oxysporum*, *F*. *tricinctum*, *F*. *solani*, *Alternaria alternata*, *Botrytis cinerea*, and *Neofusicoccum parvum*. Prior to validating the use of the primers designed for the fusariosis diagnostic, we amplified the ITS region with universal primers in order to guarantee the quality of the DNA samples used as a template (Fig 4A). As expected, the primers designed for the marker FuSp02, amplified a single band of around 590 bp on all tested *Fusarium* species but not so in the other ascomycete fungi species included in this study (Fig 4B). As a positive control, we included a previously reported marker that successfully identifies *Fusarium* species, labelled in the present study as PC01 (from Positive Control 01, Table 3 and Fig 4B) [87]. Based on the obtained results (Fig 4), we conclude that FuSp02 marker shows a high level of *Fusarium* specificity comparable to PC01. With only one exception (the *F*. *graminearum* species), the primers designed for FuSp01 marker shown only the unique expected product for the species of *Fusarium* genus which were tested. Meanwhile, the results obtained with FuSp03 marker were inconsistent, obtaining the desired product in only few cases. We concluded that FuSp02 seems to be a suitable marker for diagnosis of fusariosis because amplified efficiently using DNA from only *Fusarium* species but not from DNA of other ascomycete fungi (Fig 4).

#### 2.2. The primers designed to identify *Fusarium kuroshium* are highly efficient for the diagnosis of *Fusarium* dieback disease

**Fig 5:**
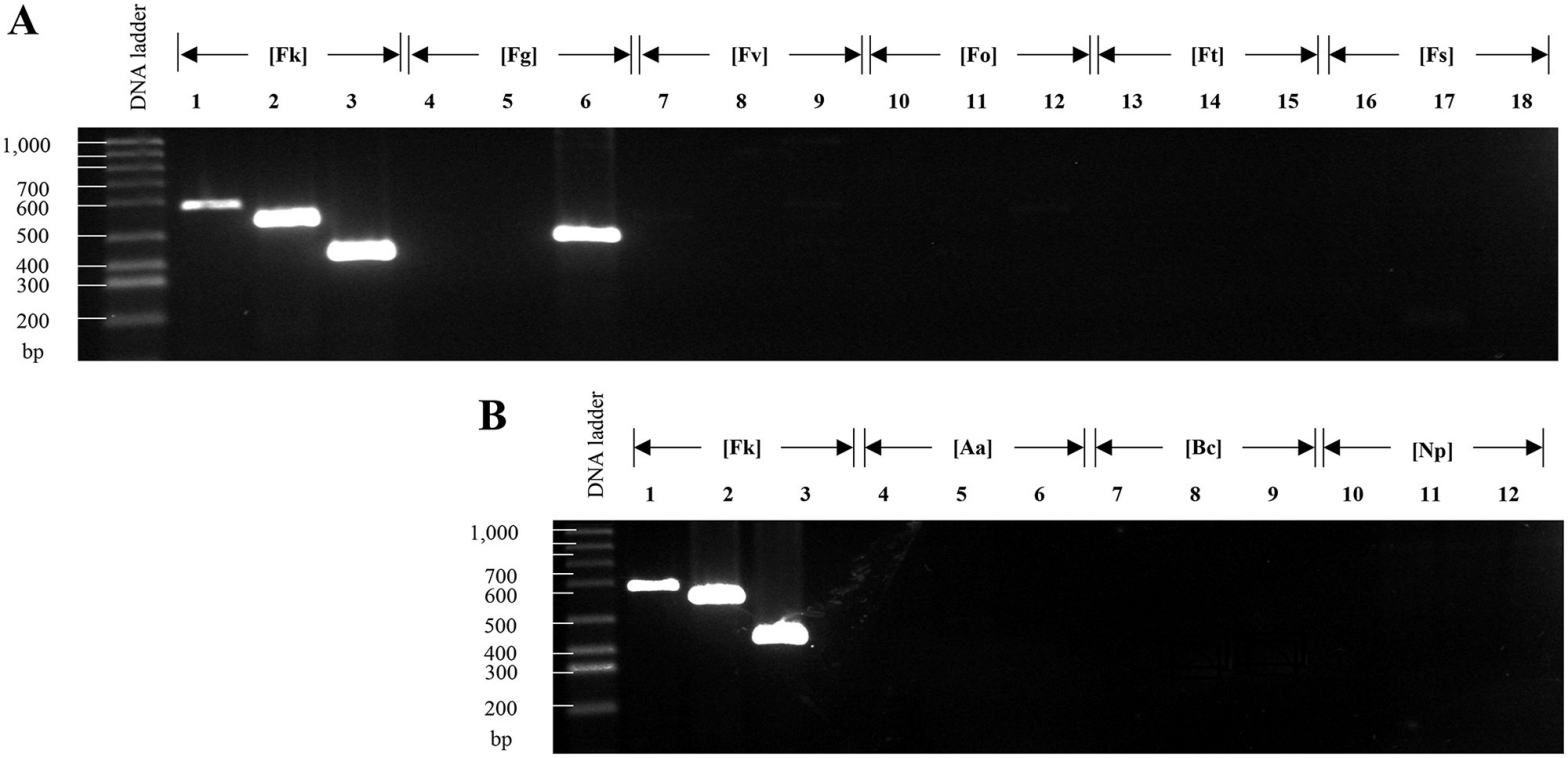
PCR analysis of the markers designed for *Fusarium kuroshium* diagnosis. From left to right, every three lanes correspond to each of the different markers (FuKu01, FuKu02, and FuKu03, respectively). These markers were tested using genomic DNA from different species of the *Fusarium* genus **(A)** and another phytopathogenic ascomycete fungi **(B)**. Tested species were: On (A), *F*. *kuroshium* ([Fk]; lanes 1–3), *F*. *graminearum* ([Fg]; lanes 4–6), *F*. *verticillioides* ([Fv]; lanes 7–9), *F*. *oxysporum* ([Fo]; lanes 10–12), *F*. *tricinctum* ([Ft]; lanes 13–15), and *F*. *solani* ([Fs]; lanes 16–18); while on (B), *Fusarium kuroshium* ([Fk]; lanes 1–3), *A*. *alternata* ([Aa]; lanes 4–6), *B*. *cinerea* ([Bc]; lanes 7–9), and *N*. *parvum* ([Np]; lanes 10–12).

*Fusarium* genus comprises a wide range of phytopathogen species [90]; and, in this study, our main goal was to design specific primers that can be used to identify the *F*. *kuroshium* specie and to generate an efficient diagnosis system for *Fusarium* dieback disease. We obtained three sets of primers that we named as FuKu01, FuKu02, and FuKu03, and which can generate 600, 599, and 466 bp amplicons, respectively. These primers were tested using genomic DNA of six different *Fusarium* species (including the species of interest, *F*. *kuroshium*) and other three species of phytopathogenic ascomycete, *A*. *alternata*, *B*. *cinerea*, and *N*. *parvum*. As shown in Fig 5A, we obtained amplicons of the expected sizes only when *F*. *kuroshium* genomic DNA was used as a template (lanes 1, 2, and 3). Markers FuKu01 and FuKu02 generate amplicons of the expected size and are species-specific. In the case of FuKu01, this genetic marker generated a noticeably lower amount of product with respect to FuKu02. As expected, these two markers fail to generate any product when using genomic DNA from other *Fusarium* species even when they are closely related to the species of interest. For example, *Fusarium* sp. associated with *X*. *morigerus* (lanes 16–18), a strain which on this study was also isolated from an ambrosia beetle species. Regarding FuKu03, this genetic marker also amplified only in the case of *F*. *graminearum* besides the species of interest (*F*. *kuroshium*). We consider that even when the amplicon obtained for *F*. *graminearum* is slightly longest with respect to the amplicon obtained for *F*. *kusoshium*, this marker (FuKu03) is not useful for the diagnosis because this difference could be related either to presence/absence of one or several introns, or maybe related to differences in the size of some of them. In principle, we do not attribute this difference to the obtaining of a non-specific product. It is important to emphasize the total absence of these three amplicons (gene markers) when template DNA belongs from other phytopathogenic ascomycetes (Fig 5B).

**Fig 6:**
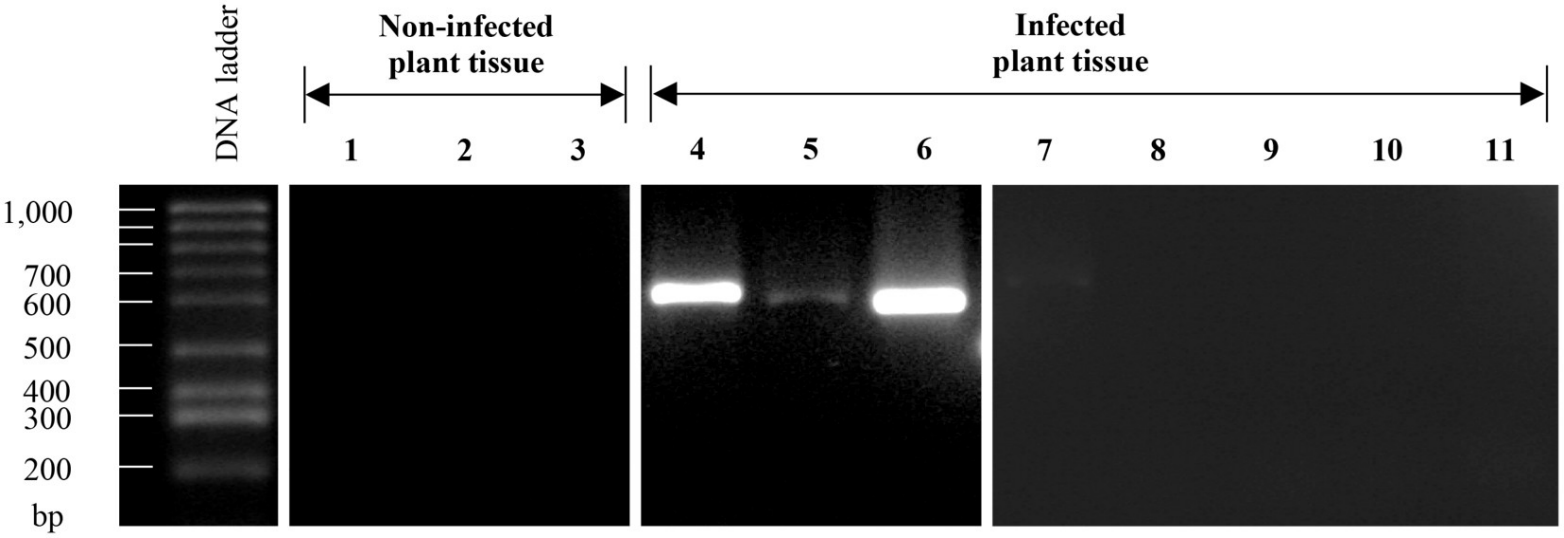
Evaluation of the diagnosis system using as template DNA obtained from a plant tissue artificially infected tissue with *F*. *kuroshium*. This test included the FuSp02 genus-specific marker and FuKu01 and FuKu02 species-specific markers. Lanes 1, 2 and 3 which showed a total absence of any amplicons correspond to negative controls, that is, DNA used as template was isolated from non-infected plant tissue. For the rest of the lines, DNA used as template comes from plant tissue artificially infected tissue with *F*. *kuroshium*. Lane 4 represents FuSp02, while lanes 5 and 6 show the expected products for markers FuKu01 and FuKu02. As additional controls, markers designed for *F*. *graminearum* were also tested. Two of them designed in the present study (FuGr01 and FuGr02; lanes 7 and 8), and the other one (PC02; lane 9), previously reported [88]. In the same way, the FuOx01 and FuOx02 markers designed on this study for *F*. *oxysporum*, were also tested (lanes 10 and 11). Additional results regarding markers designed on this study to *F*. *graminearum* and *F*. *oxysporum* are shown in sections downstream described.

Next, using DNA isolated from *Persea americana* cv. Hass stalks artificially infected with *F*. *kuroshium* (see S1 Fig and Methods for more details), we tested the reliability of the primers designed to diagnose both, fusariosis (genus-specific primers) and the *Fusarium* dieback disease (primers designed specifically for *F*. *kuroshium*). Consistent with the results presented above, FuSp02 genetic marker was successfully used to amplify the expected fragment, confirming the presence of genetic material in the DNA sample which belongs to one (or several) *Fusarium* species. To define the species, FuKu01 and Fuku02 genetic markers were successfully used (Fig 6).

**Fig 7:**
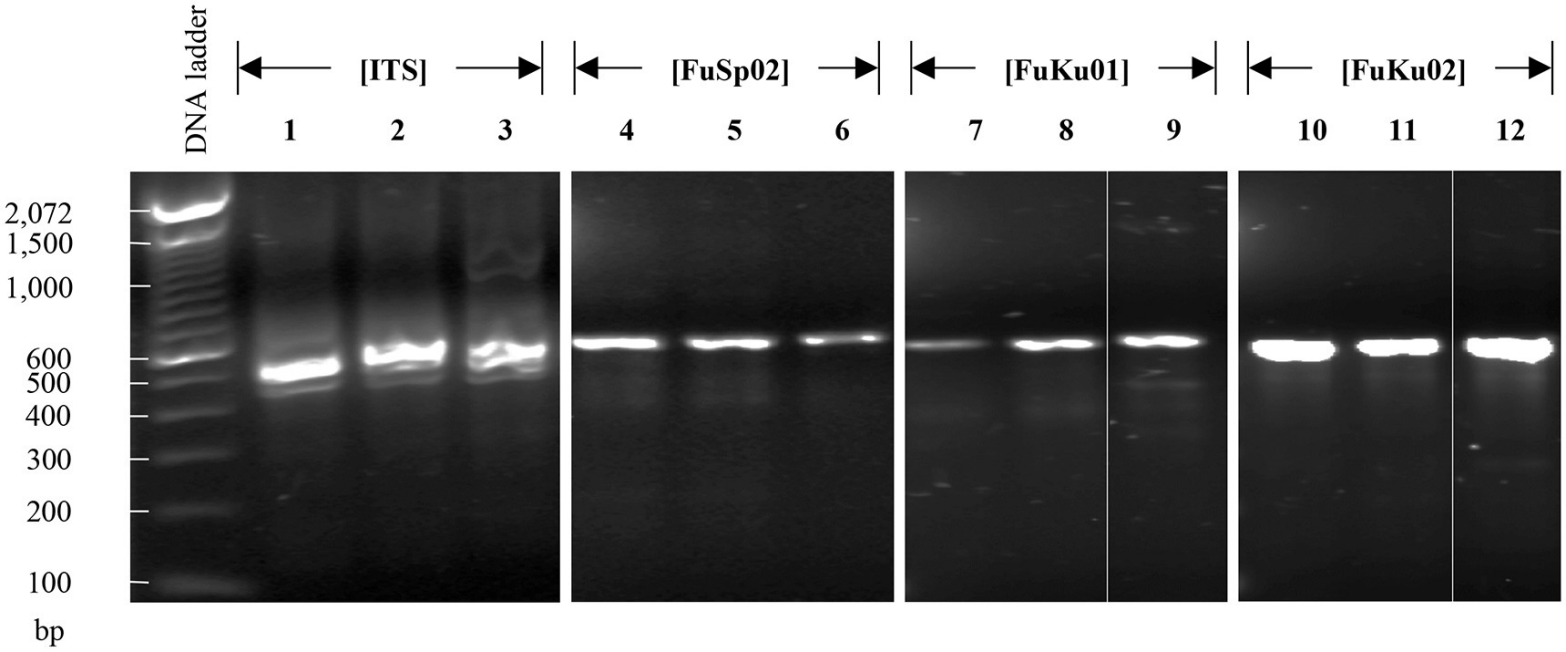
Evaluation of the diagnosis system using as template the DNA isolated from plant tissue of *E*. *corallodendron* trees, which were collected in the field and it was infested by KSHB and infected with *F*. *kuroshium*. Lanes 1–3 show the amplicons corresponding to ITS, one lane per independently collected tree. Lanes 4–6, FuSp02 marker (genus-specific). Lanes 7–9, and 10–12, species-specific markers designed for *F*. *kuroshium* (FuKu01 and Fuku02).

We also test the effectiveness of the primers designed for fusariosis diagnostic on field samples. In our search of symptomatic trees, only three trees of the *Erythrina corallodendron* species were identified in a unique and small location (S2 Fig and S3 Table, see Methods for more details). First, to confirm the presence of genetic material from fungi on the DNA isolated from infected plant tissue, we amplify the ITS region (Fig 7, lanes 1–3). Then, using a genus-specific marker (FuSp02) we confirm that this fungus (or fungi) belongs to the *Fusarium* genus (Fig 7, lanes 4–6). Finally, FuKu01 and FuKu02 successfully identified the pathogen *F*. *kuroshium* (Fig 7, lanes 7–12). Equal that *in vitro* assays, primers designed to amplify specifically other species belonging to *Fusarium* genus (*F*. *graminearun* and *F*. *oxysporum*), were also used as negative controls and as expected, with none of them we obtained PCR products (data not shown).

#### 2.3. The strategy of pan-genome analysis allows the identification of specific markers for the different species which involved in fusariosis

**Fig 8:**
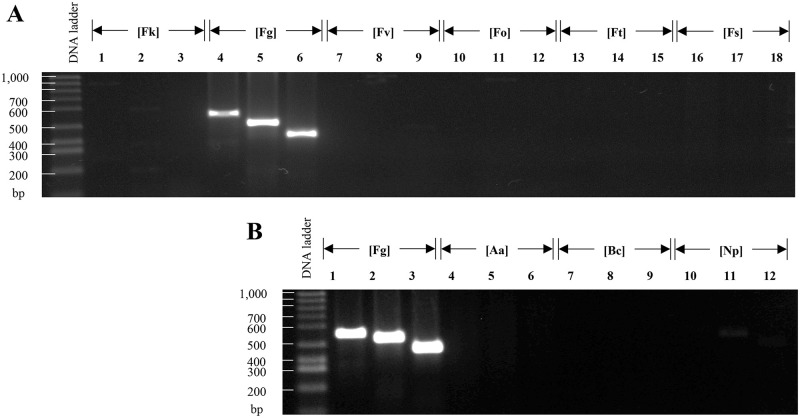
PCR analysis of primers designed for *Fusarium graminearum*. From left to right, every third lane corresponds to the markers (FuGr01, FuGr02, and PC02) tested using genomic DNA from different species of the *Fusarium* genus and other phytopathogenic ascomycete fungi. **(A)** Lanes 1–3 correspond to *Fusarium kuroshium* [Fk], 4–6 to *F*. *graminearum* [Fg], 7–9 to *F*. *verticillioides* [Fv], 10–12 to *F*. *oxysporum* [Fo], 13–15 to *F*. *tricinctum* [Ft], and 16–18 to *F*. *solani* [Fs]; while on **(B)**, lanes 1–3 correspond to *F*. *graminearum* [Fg], 4–6 to *Alternaria alternata* [Aa], 7–9 to *Botrytis cinereal* [Bc], and 10–12 to *N*. *parvum* [Np].

In order to provide additional evidence of the suitability of the application of pan-genome analysis for select markers and design primers to be used in a PCR-based diagnosis system, we evaluate the primers designed for the detection of *F*. *graminearum* and *F*. *oxysporum*. The primers designed for these species showed specificity, because only amplified DNA of the species for which they were designed. As a result of our analyses, we proposed two markers as diagnostic tools for *F*. *graminearum* (FuGr01 and FuGr02, Table 3); these allowed for the generation of 549 and 509 bp amplicons, respectively. We used as a positive control the primers previously reported to detect the presence of strains of the *F*. *graminearum* species ([88], see Table 3, PC02). After the analysis of band patterns obtained by electrophoresis, it was evident that primers designed on this study as well as positive control were specific for *F*. *graminearum*; these primers produced no unspecific products, neither with other species of the genera or with other ascomycetes (Fig 8).

**Fig 9:**
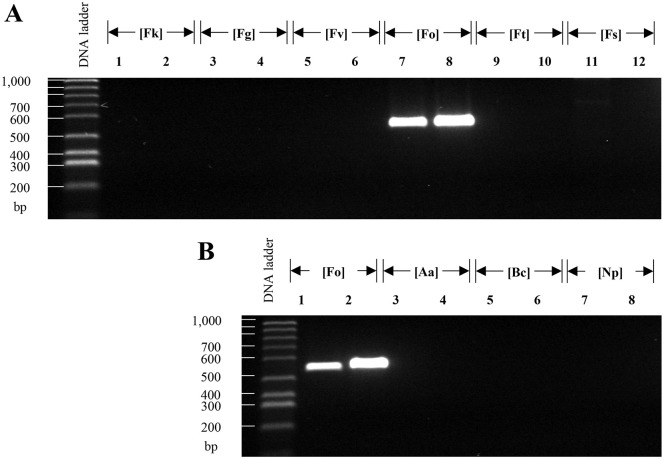
Evaluation of primers designed for the diagnosis of *F*. *oxysporum*. From left to right, every two lanes correspond to the markers FuOx01, FuOx02. In **(A)**, lanes 1 and 2 correspond to *Fusarium kuroshium* [Fk], 3 and 4 to *F*. *graminearum* [Fg], 5 and 6 to *F*. *verticillioides* [Fv], 7 and 8 to *F*. *oxysporum* [Fo], 9 and 10 to *F*. *tricinctum* [Ft], and 11 and 12 to *F*. *solani* [Fs]. In **(B)**, lanes 1 and 2 showed the PCR products of *F*. *oxysporum* [Fo], 3 and 4 of *Alternaria alternata* [Aa], 5 and 6 from *Botrytis cinereal* [Bc], and 7 and 8 of *N*. *parvum* [Np].

**Fig 10:**
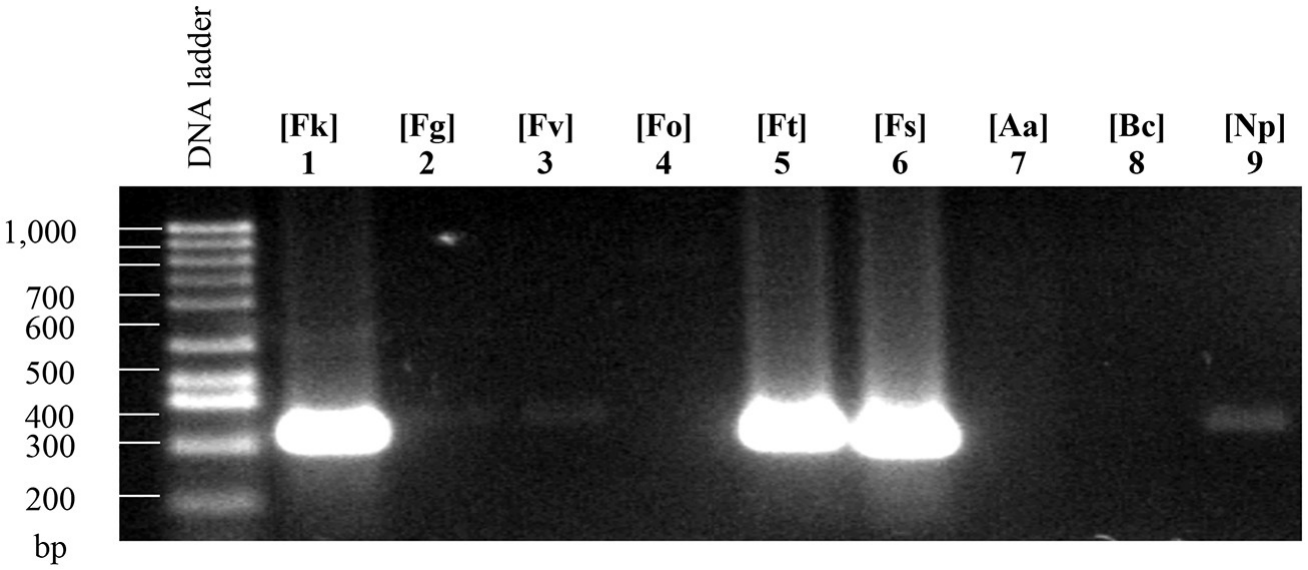
PCR analysis of the PC03 marker previously reported for the diagnosis of *F*. *oxysporum*. From left to right are shown the PCR products obtained for *Fusarium kuroshium* [Fk], *F*. *graminearum* [Fg], *F*. *verticillioides* [Fv], *F*. *oxysporum* [Fo], *F*. *tricinctum* [Ft], *F*. *solani* [Fs], *A*. *alternata* [Aa], *B*. *cinereal* [Bc], and *N*.*parvum* [Np], lanes 1 to 9, respectively.

For the diagnosis of *F*. *oxysporum* we identified two possible markers candidates, FuOx01 and FuOx02. Primers designed to amplify these markers produced amplicons with 554 and 556 bp sizes, respectively. The experimental validation using genomic DNA from the strain reveals high specificity (Fig 9). We use a previously reported set of primers as a positive control ([89], Table 3, PC03); however, in contrast with the high degree of specificity of the markers proposed in this study, the positive control of *F*. *oxysporum* (PC03) amplified PCR products of 280 pb when DNA used as template was isolated from *Fusarium kuroshium*, *F*. *solani*, *F*. *graminearum*, *F*. *verticillioides*, and *N*. *parvum* (Fig 10, lanes 1, 2, 3, 6 and 9, respectively). Which indicates that the primers previously reported are not useful to identify a species-specific marker that can be used to diagnose fusariosis caused by strains of *F*. *oxysporum* species.

## Conclusions

In this study, we identify highly divergent coding regions, and we prove that they can be used to design specific primers that allow the identification of genus- or specie- in specific manner (in this study we mainly focused our searches on species from *Fusarium* genus). Based on presented results we propose that this method could be employed to design primers or probes for other pathogenic species or strains. We confirmed by validating species-specific and genera specific genes of at least three different species of the *Fusarium* spp., that the proposed method is capable to identify reliable marker. We also prove the technique is stable enough to prevent erroneous diagnoses even when dealing with closely related species (e.g., *F*. *solani* and *F*. *kuroshium*).

A really low percent of the designed primers under proposed methodology can generate inconsistent results, we consider that this problem can be associated with automatic gene prediction algorithms often make errors and can jeopardize subsequent analyses. This issue obviously could be more frequent in low quality draft genomes with complex gene structures and assembled with low coverages. These low-quality genomes could be no included, however, it should be considered that increasing the number of genomes in pangenome analysis, it undoubtedly increases the reliability with which specific genus or species markers are identified. Based on the above, we suggest that gene model prediction processes need to be accompanied by the implementation of some dedicated computational methods to correct mistakes from the predictions and accompanied by some manual curation.

KSBH is considered a phytosanitary problem of national relevance in Mexico. The advantage of performing the PCR analysis directly from the DNA extraction of infected tissue *in-vitro* and *in-vivo* is a key innovation of the diagnosis system. Therefore, it is not necessary to isolate and grow the fungus. Our results inferred that the system may function at early infection stages however it is important to consider the use of and effective sampling method.

Short-term availability of the system is expected to be provided as a kit including not only the essential primers but also control DNA to be generated after cloning the amplicons generated for each of the proposed markers. Thus, users of this tool are guaranteed to have positive controls always available to bring complete certainty to their diagnosis. Finally, is important to consider that our system is susceptible to adapt to qPCR to become a more sensitive method.

## Supporting information

S1 Fig *Fusarium kuroshium* inoculations in avocado (*Persea americana* cv. Hass) stalks. (PDF)

S2 Fig Collected field samples. (PDF)

S1 Table Overview of proteomes used for pan-genome analysis. This table showed the name of the strains from where the genomic information was obtained, as well as the metadata associated.
(XLSX)

S2 Table List of proteomes used on the database for gene model prediction. (XLSX)

S3 Table Field sampled sites location of infested trees. The first column described the native name, second the scientific name, and finally the georeferenced sites of the sampled sites.
(XLSX)

S1 Raw images (PDF)
