# Supplementary material for: Design of a diagnostic system based on molecular markers derived from the ascomycetes pan-genome analysis: The case of Fusarium dieback disease
Source: PLoS One. 2021 Jan 28;16(1):e0246079. doi: 10.1371/journal.pone.0246079 (PMC7843019; doi:10.1371/journal.pone.0246079)
Supplement: S1 Fig — (PDF) [file pone.0246079.s001.pdf]

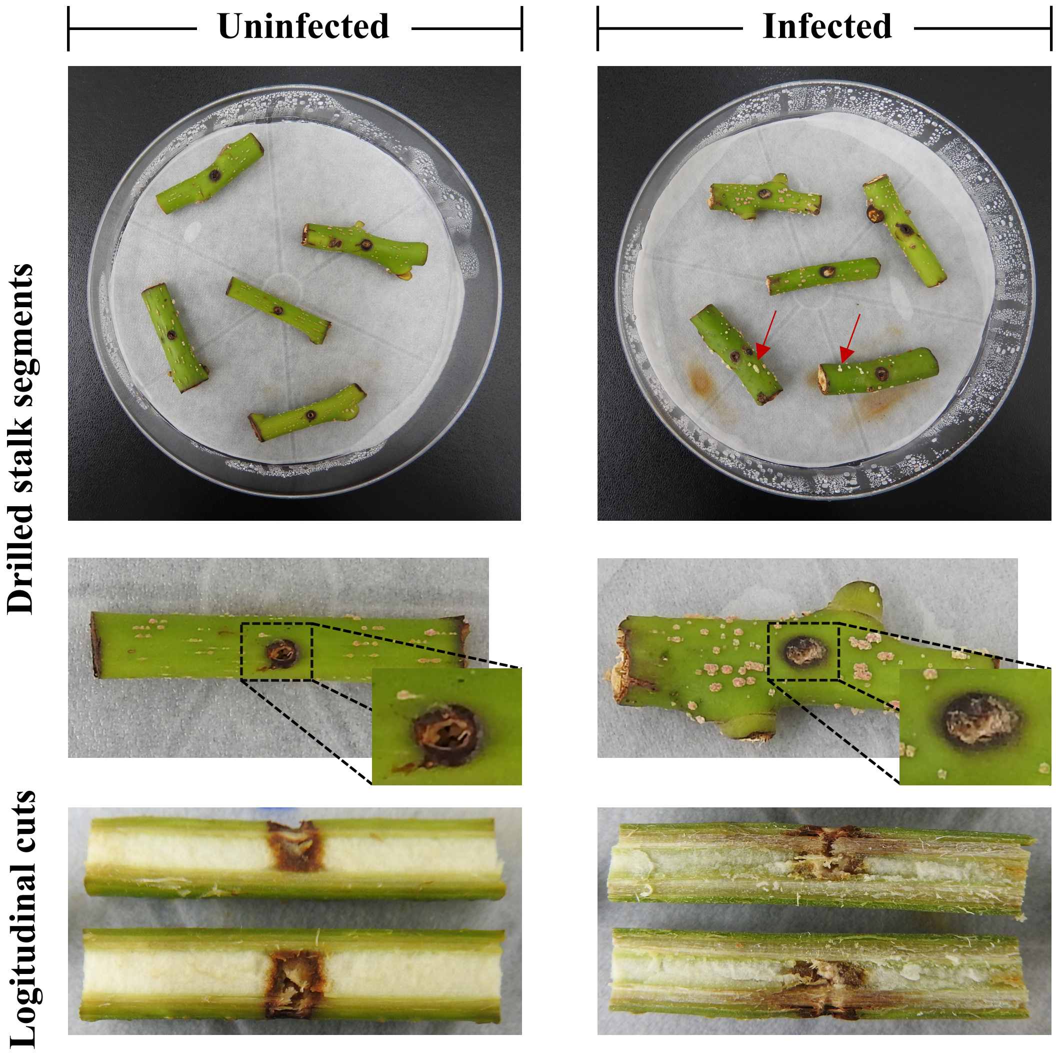


**S1 Figure.** ***Fusarium kuroshium*** **inoculations in avocado (*Persea americana* cv. Hass) stalks.** The photographs show the stalk segments uninfected (left) and infected (rigth) at 7 days post inoculation. At this time is clearly distinguish a white exudate in the infected stalks surface (red arrows) that is scarce in control stalks. In addition, the uninfected stalks displayed a response associated with the injury showing a brownish zone around the mechanically damaged, but the zone did not extend more than 0.2-0.4 mm. In contrast, the zone of the inoculation site of the infected stalks segments turned black. The symptoms are more evident on longitudinal cuts which also showed the necrosis progression. The photographs were taken with a stereoscopic microscope. Photographs of distinct stages of disease progression can be consulted in a related work (Perez-Torres C.A. & Ibarra-Laclette E. *et al.*, in review).
