# Supplementary material for: Design of a diagnostic system based on molecular markers derived from the ascomycetes pan-genome analysis: The case of Fusarium dieback disease
Source: PLoS One. 2021 Jan 28;16(1):e0246079. doi: 10.1371/journal.pone.0246079 (PMC7843019; doi:10.1371/journal.pone.0246079)
Supplement: S2 Fig — (PDF) [file pone.0246079.s002.pdf]

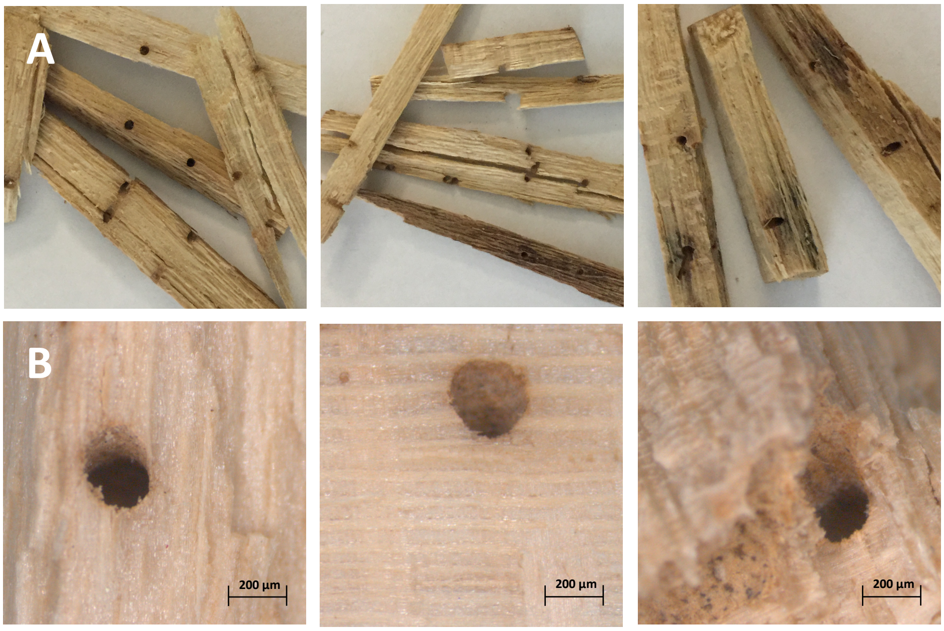

S2 Figure. Collected field samples. A. Wood chips recovered from each of three distinct *Erythrina corallodendron* trees which showed obvious symptoms of the Fusarium dieback disease. **B.** Close-up of the drilled galleries by KSHB.
