# Supplementary material for: Design of a diagnostic system based on molecular markers derived from the ascomycetes pan-genome analysis: The case of Fusarium dieback disease
Source: PLoS One. 2021 Jan 28;16(1):e0246079. doi: 10.1371/journal.pone.0246079 (PMC7843019; doi:10.1371/journal.pone.0246079)

Fig. 4 (Figure legend).

PCR-based assays using FuSp02 and PC01 [87] as diagnostic markers of fusariosis. Agarose (0.8%) gels electrophoresis which shows: **(A)** The amplified ITS region to validate the quality of the template DNA used and isolated from different species of ascomycetes fungi (*F. kuroshium* [Fk], *F. graminearum* [Fg], *F. verticillioides* [Fv], *F. oxysporum* [Fo], *F. tricinctum* [Ft], *F. solani* [Fs], *A. alternata* [Aa], *B. cinereal* [Bc], and *Neofusicoccum parvum* [Np]; lines 1-9, respectively), and **(B)** The PCR fragments (amplicons) obtained for FuSp02 (odd lanes) and PC01 (even lanes) markers, both of them highly-specific to identify species from *Fusarium* genus.

Notes.

The photographs were taken with Gel DocTM XR+ Imaging system, using Image Lab softwareTM (BIORAD). The red line box represents the gel area used to generate the figure. Brightness and contrast were slightly-adjusted on the entire image. This process NO misrepresent any results or information discussed in the main text.

Fig. 4 (A panel).

Original image

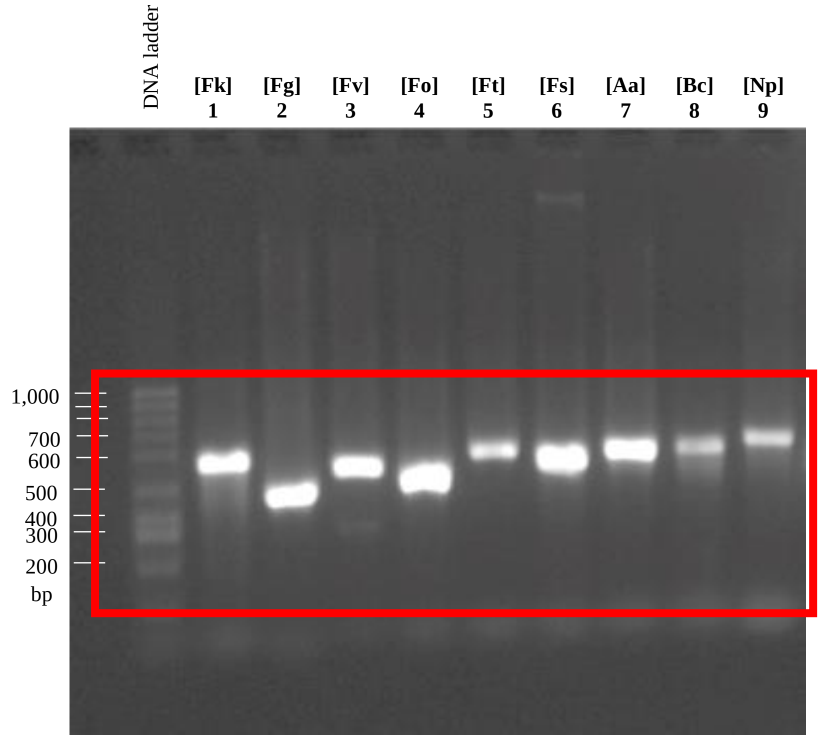

Generated image

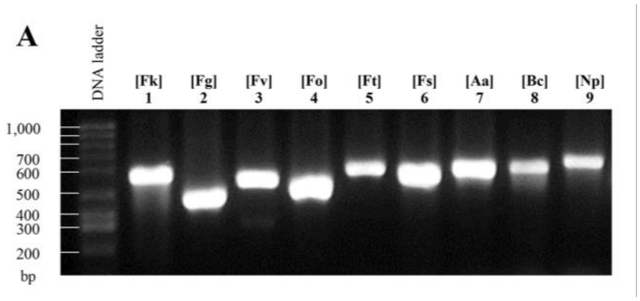

Fig. 4 (B panel).

Original image

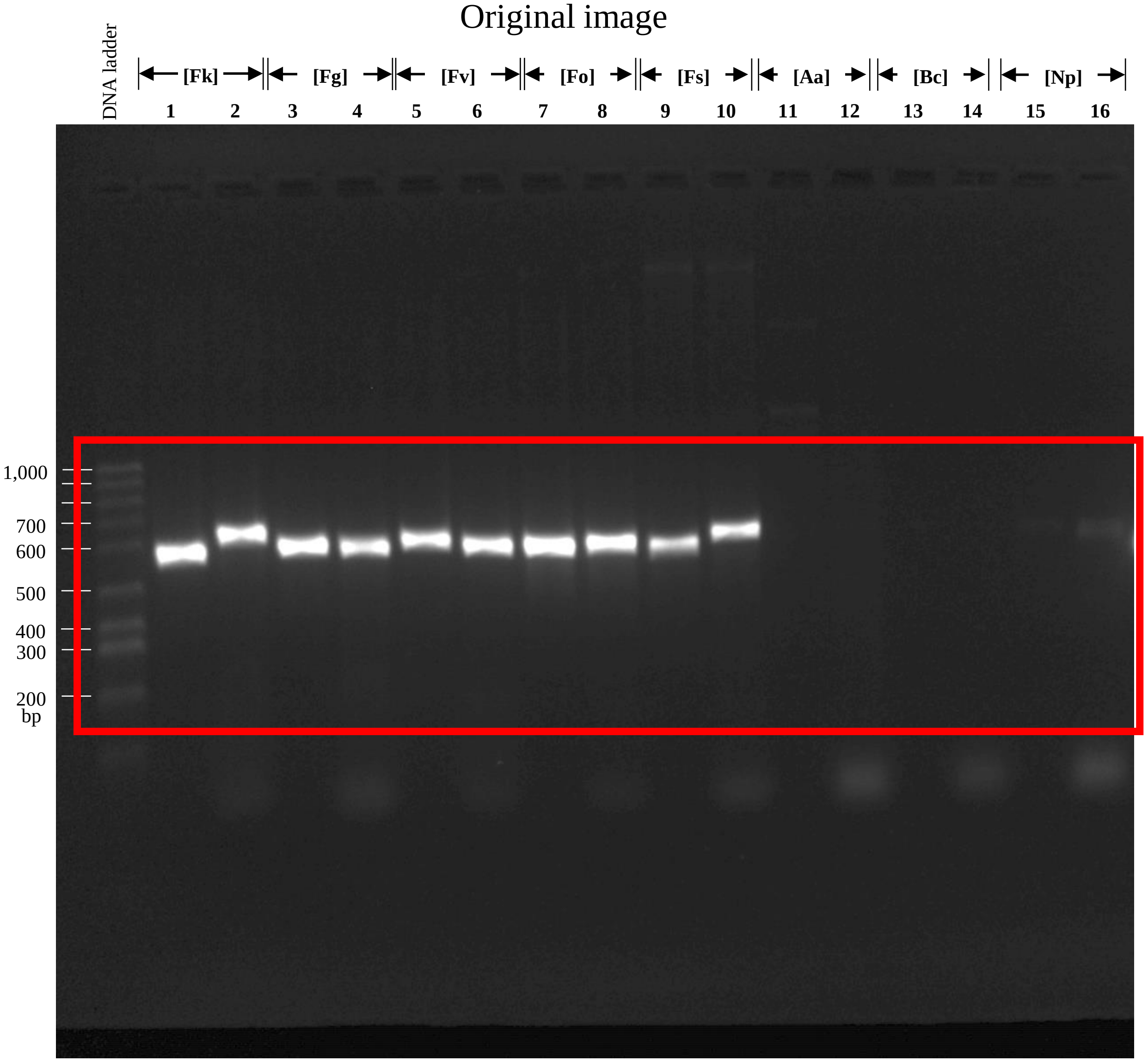

Generated image

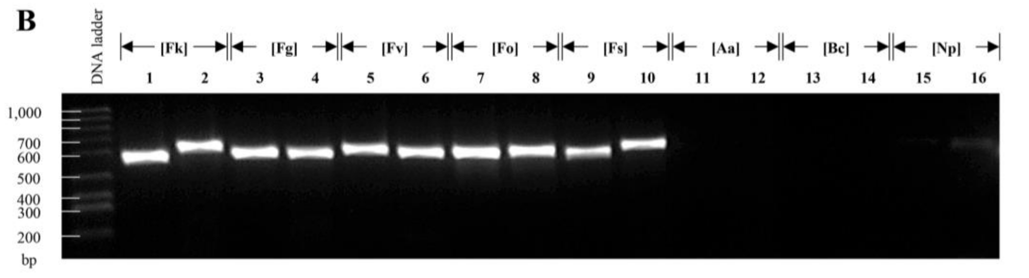

Fig. 5 (Figure legend).

PCR analysis of the markers designed for *Fusarium kuroshium* diagnosis. From left to right, every three lanes correspond to each of the different markers (FuKu01, FuKu02, and FuKu03, respectively). These markers were tested using genomic DNA from different species of the *Fusarium* genus **(A)** and another phytopathogenic ascomycete fungi **(B)**. Tested species were: On (A), *F. kuroshium* ([Fk]; lanes 1-3), *F. graminearum* ([Fg]; lanes 4-6), *F. verticillioides* ([Fv]; lanes 7-9), *F. oxysporum* ([Fo]; lanes 10-12), *F. tricinctum* ([Ft]; lanes 13-15), and *F. solani* ([Fs]; lanes 16-18); while on (B), *Fusarium kuroshium* ([Fk]; lanes 1-3), *A. alternata* ([Aa]; lanes 4-6), *B. cinerea* ([Bc]; lanes 7-9), and *N. parvum* ([Np]; lanes 10-12).

Notes.

The photographs were taken with Gel DocTM XR+ Imaging system, using Image Lab softwareTM (BIORAD). The red line box represents the gel area used to generate the figure. Brightness and contrast were slightly-adjusted on the entire image. This process NO misrepresent any results or information discussed in the main text or obtained as result of agarose gel electrophoresis. Lanes not included in the final figure were marked with an “X”.

Fig. 5 (A panel).

Original image

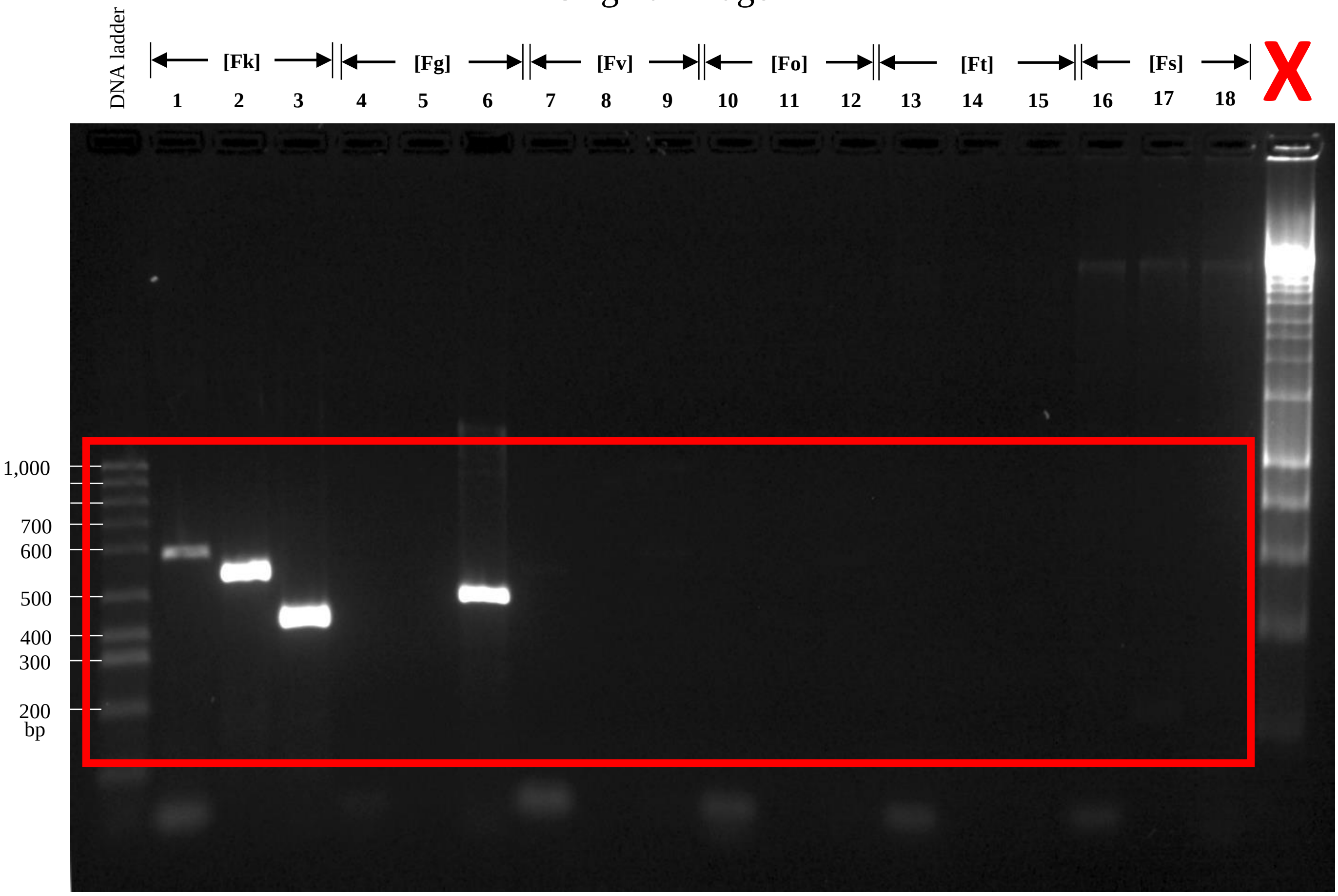

Generated image

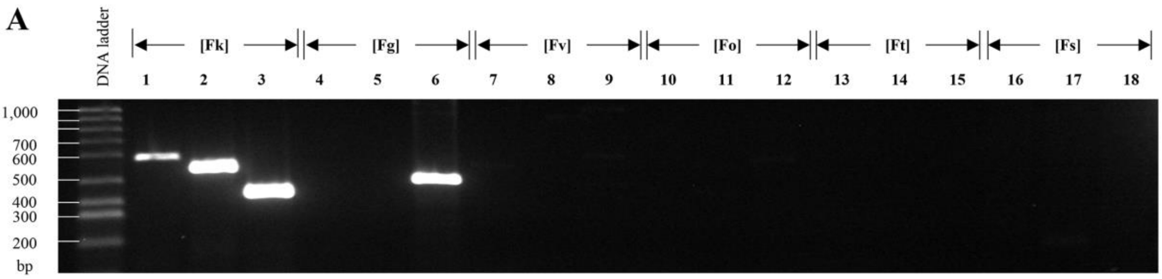

Fig. 5 (B panel).

Original image

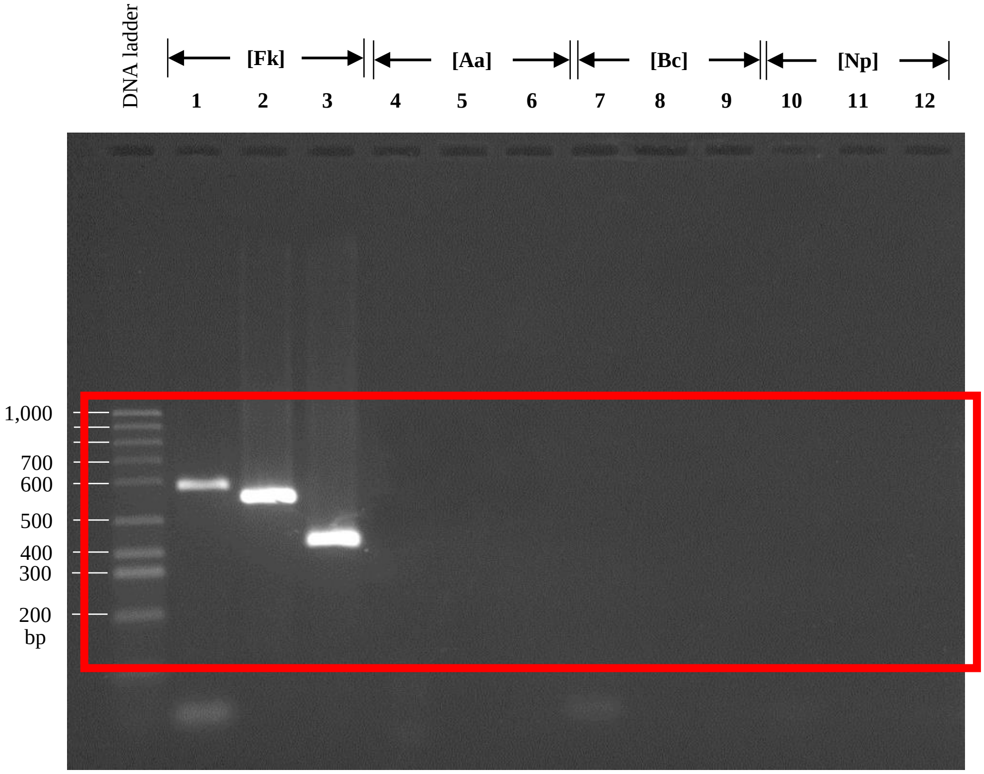

Generated image

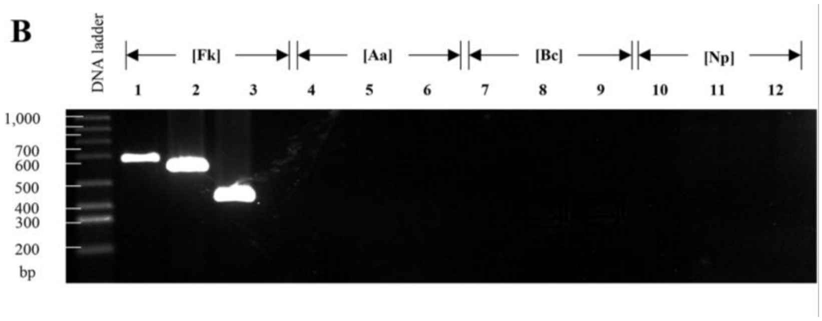

Fig. 6 (Figure legend).

Evaluation of the diagnosis system using as template DNA obtained from a plant tissue artificially infected tissue with *F. kuroshium*. This test included the FuSp02 genus-specific marker and FuKu01 and FuKu02 species-specific markers. Lanes 1, 2 and 3 which showed a total absence of any amplicons correspond to negative controls, that is, DNA used as template was isolated from non-infected plant tissue. For the rest of the lines, DNA used as template comes from plant tissue artificially infected tissue with *F. kuroshium*. Lane 4 represents FuSp02, while lanes 5 and 6 show the expected products for markers FuKu01 and FuKu02. As additional controls, markers designed for *F. graminearum* were also tested. Two of them designed in the present study (FuGr01 and FuGr02; lanes 7 and 8), and the other one (PC02; lane 9), previously reported [88]. In the same way, the FuOx01 and FuOx02 markers designed on this study for *F. oxysporum*, were also tested (lanes 10 and 11). Additional results regarding markers designed on this study to *F. graminearum* and *F. oxysporum* are shown in sections downstream described.

Notes.

Fig. 6 consists of four sections (1-4), each of them represent lanes from two distinct gels images. Sections were rearranged but the splices are clearly denoted by vertical white lines. The photographs were taken with Gel DocTM XR+ Imaging system, using Image Lab softwareTM (BIORAD). The red line box represents the area of the gel used to generate the figure. Brightness and contrast were slightly-adjusted on the entire image. This process NO misrepresent any results or information discussed in the main text. Lanes not included in the final figure were marked with an “X”.

Fig. 6 (Section 2).

Original image

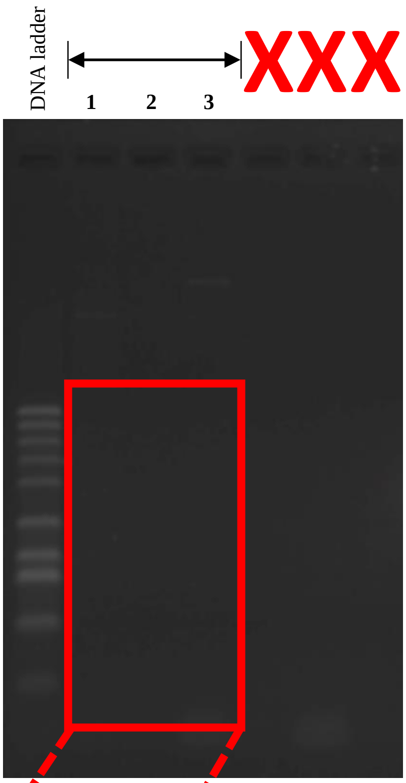

Generated image

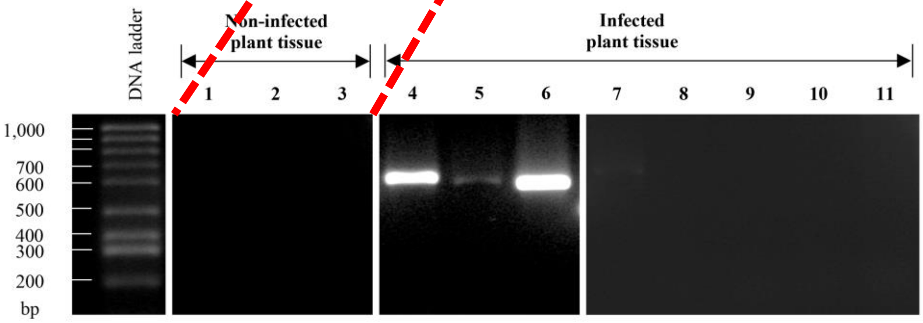

Fig. 6 (Sections 1, 3 and 4).

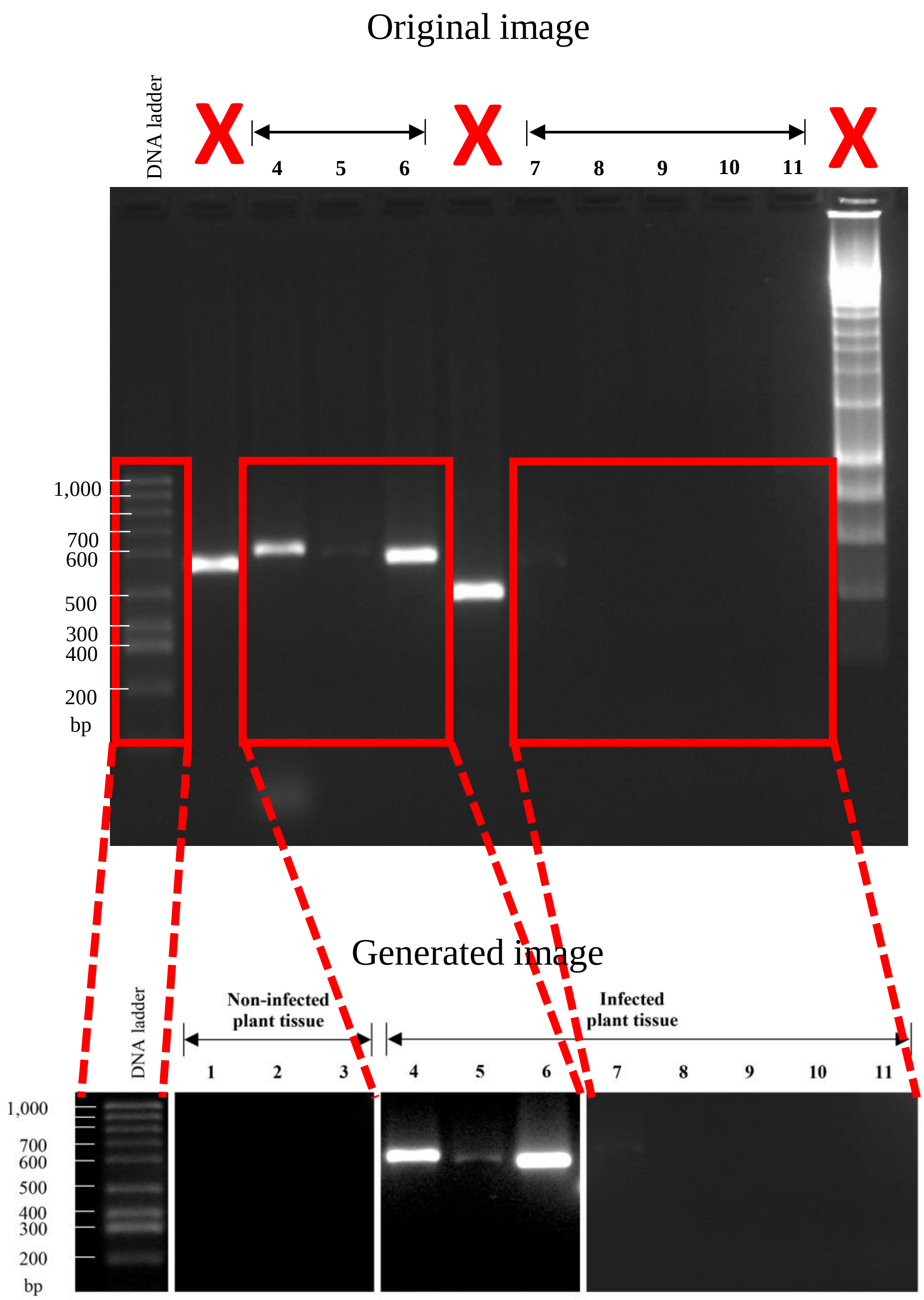

Fig. 7 (Figure legend).

Evaluation of the diagnosis system using as template the DNA isolated from plant tissue of *E. corallodendron* trees which were collected in the field and it was infested by KSHB and infected with *F. kuroshium*. Lanes 1-3 show the amplicons corresponding to ITS, one lane per independently collected tree. Lanes 4-6, FuSp02 marker (genus-specific). Lanes 7-9, and 10-12, species-specific markers designed for *F. kuroshium* (FuKu01 and Fuku02).

Notes.

Fig. 7 consists of four sections (1-4), each of them represent lanes from four distinct gel images which were used to create the figure. Sections were rearranged but the splices are clearly denoted by vertical white lanes. Fine and thick lines represent that comes from the same or distinct gel, respectively. The photographs were taken with Gel DocTM XR+ Imaging system, using Image Lab softwareTM (BIORAD). The red line box represents the area of the gel used to generate the figure. Brightness and contrast were slightly-adjusted on the entire image. This process NO misrepresent any results or information discussed in the main text or obtained as result of agarose gel electrophoresis. Lanes not included in the final figure were marked with an “X”.

Fig. 7 (Sections 1 and 2).

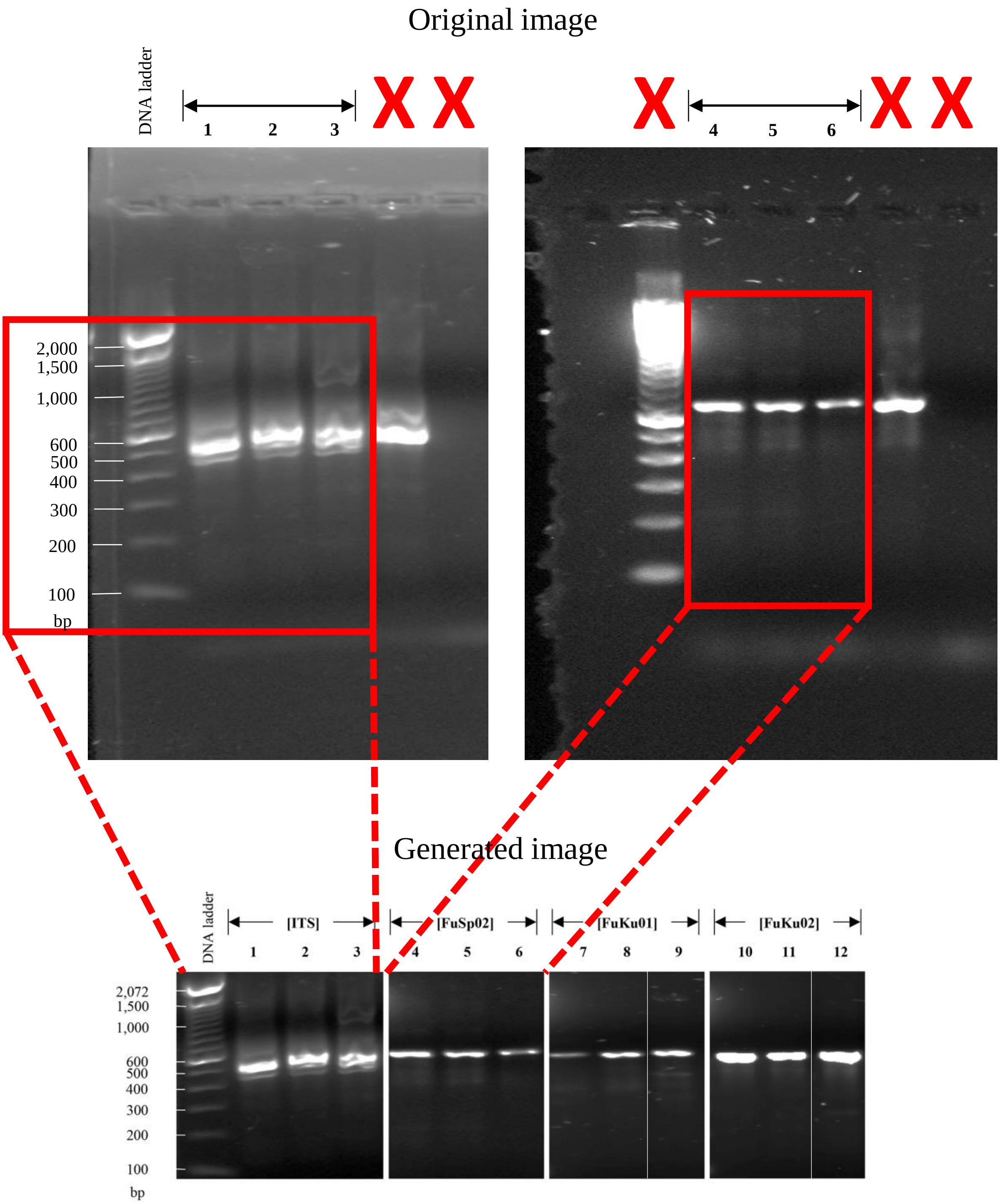

Fig. 7 (Sections 3 and 4).

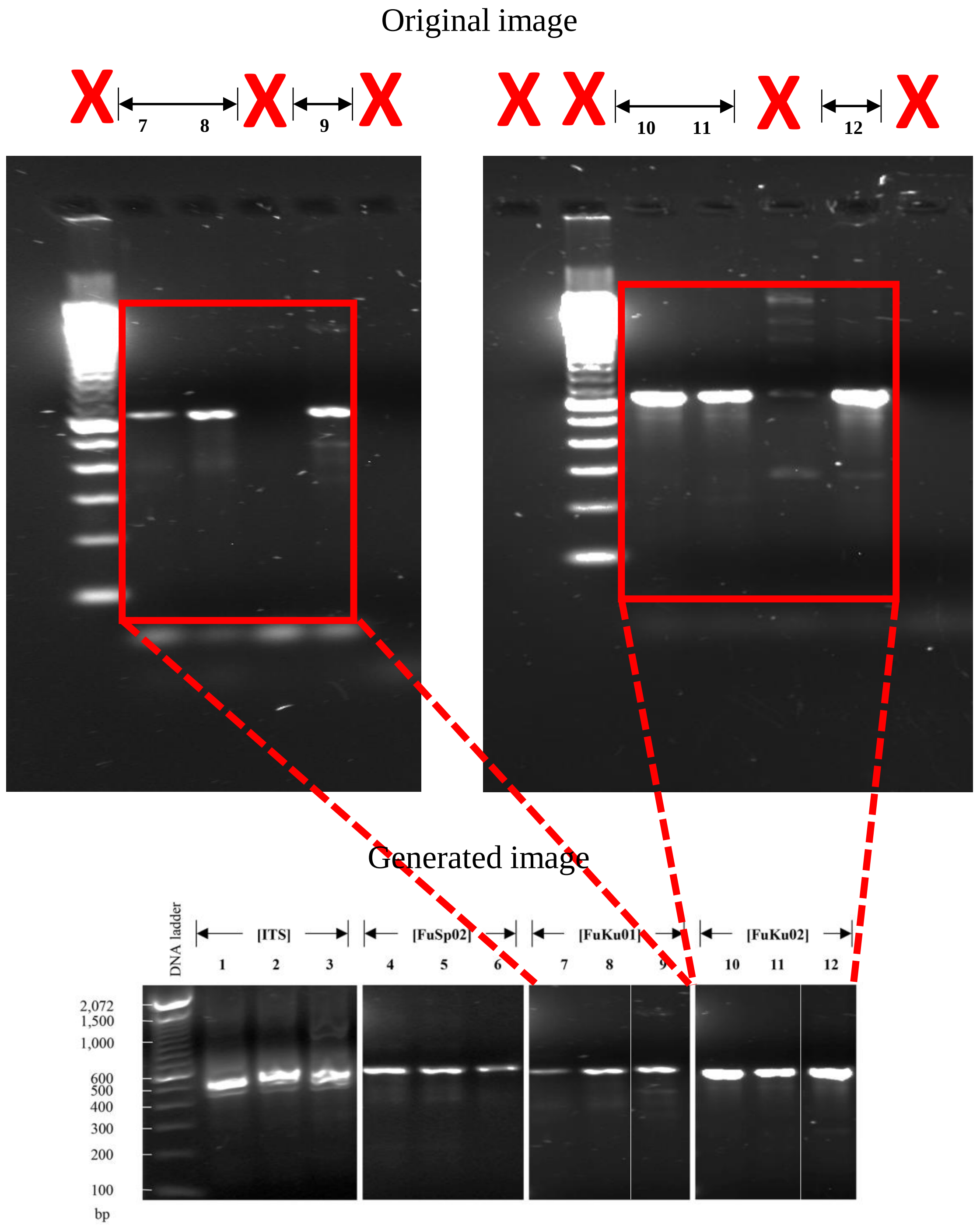

Fig. 8 (Figure legend).

PCR analysis of primers designed for *Fusarium graminearum*. From left to right, every third lane corresponds to the markers (FuGr01, FuGr02, and PC02) tested using genomic DNA from different species of the *Fusarium* genus and other phytopathogenic ascomycete fungi. **(A)** Lanes 1-3 correspond to *Fusarium kuroshium* [Fk], 4-6 to *F. graminearum* [Fg], 7-9 to *F. verticillioides* [Fv], 10-12 to *F. oxysporum* [Fo], 13-15 to *F. tricinctum* [Ft], and 16-18 to *F. solani* [Fs]; while on **(B)**, lanes 1-3 correspond to *F. graminearum* [Fg], 4-6 to *Alternaria alternata* [Aa], 7-9 to *Botrytis cinereal* [Bc], and 10-12 to *N. parvum* [Np].

Notes.

The photographs were taken with Gel DocTM XR+ Imaging system, using Image Lab softwareTM (BIORAD). The red line box represents the gel area used to generate the figure. Brightness and contrast were slightly-adjusted on the entire. This process NO misrepresent any results or information discussed in the main text or obtained as result of agarose gel electrophoresis. Lanes not included in the final figure were marked with an "X".

Fig. 8 (A panel).

Original image

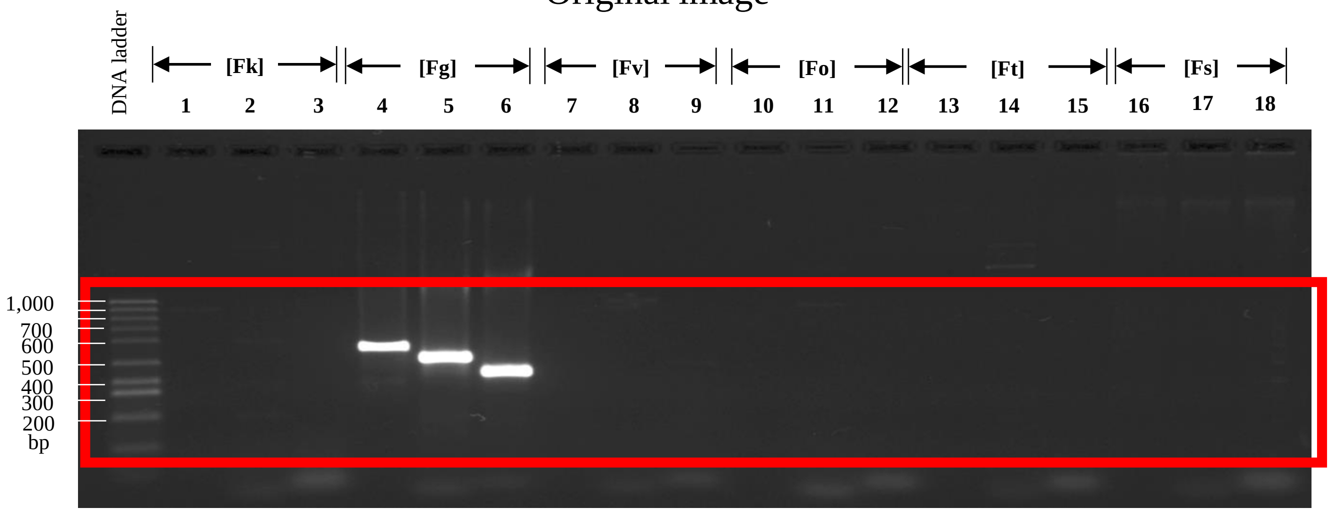

Generated image

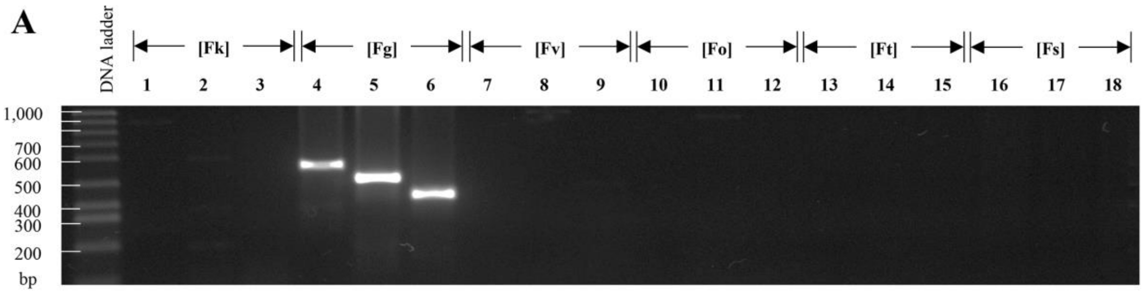

Fig. 8 (B panel).

Original image

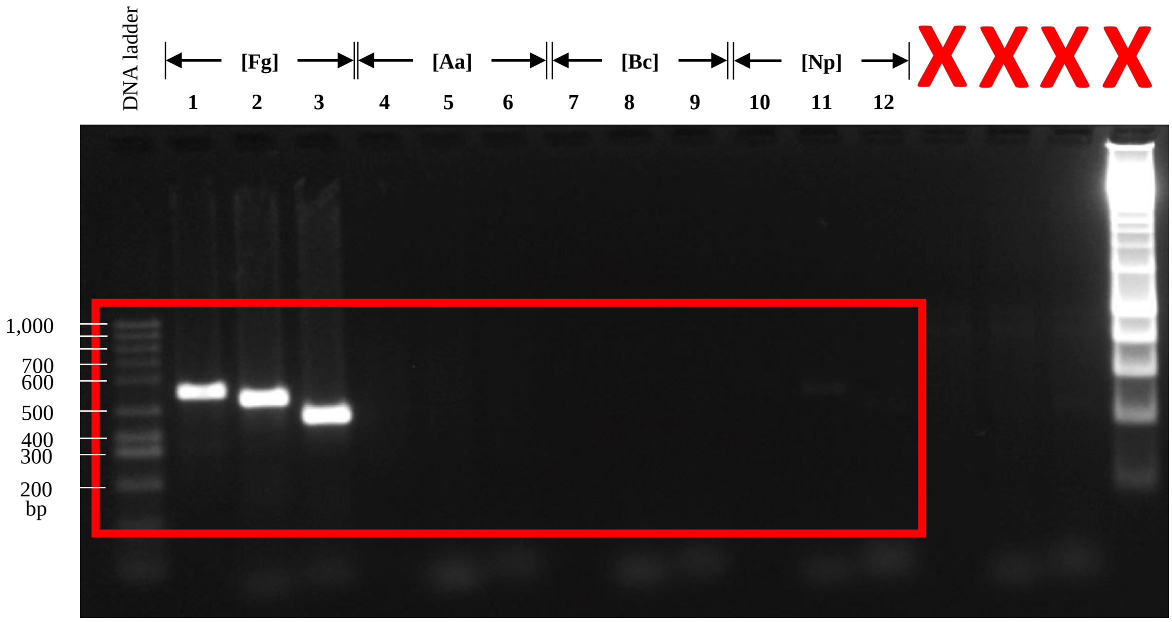

Generated image

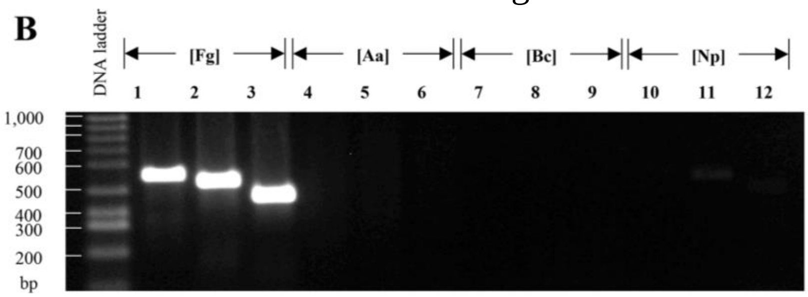

Fig. 9 (Figure legend).

Evaluation of primers designed for the diagnosis of *F. oxysporum*. From left to right, every two lanes correspond to the markers FuOx01, FuOx02. In **(A)**, lanes 1 and 2 correspond to *Fusarium kuroshium* [Fk], 3 and 4 to *F. graminearum* [Fg], 5 and 6 to *F. verticillioides* [Fv], 7 and 8 to *F. oxysporum* [Fo], 9 and 10 to *F. tricinctum* [Ft], and 11 and 12 to *F. solani* [Fs]. In **(B)**, lanes 1 and 2 showed the PCR products of *F. oxysporum* [Fo], 3 and 4 of *Alternaria alternata* [Aa], 5 and 6 from *Botrytis cinereal* [Bc], and 7 and 8 of *N. parvum* [Np].

Notes.

The photographs were taken with Gel DocTM XR+ Imaging system, using Image Lab softwareTM (BIORAD). The red line box represents the gel area used to generate the figure. Brightness and contrast were slightly-adjusted on the entire image. This process NO misrepresent any results or information discussed in the main text or obtained as result of agarose gel electrophoresis. Lanes not included in the final figure were marked with an "X".

Fig. 9 (A panel).

Original image

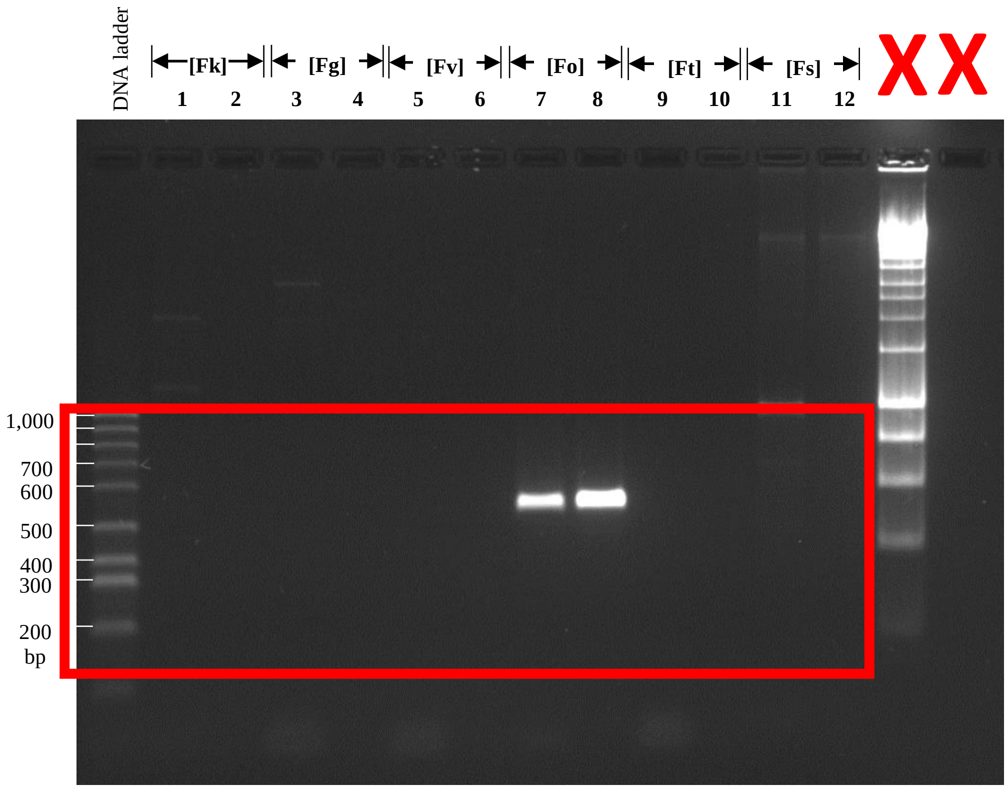

Generated image

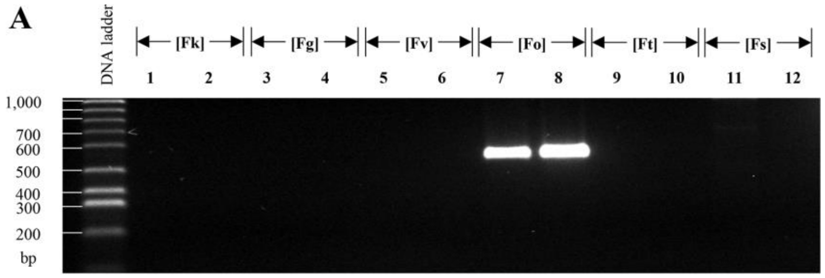

Fig. 9 (B panel).

Original image

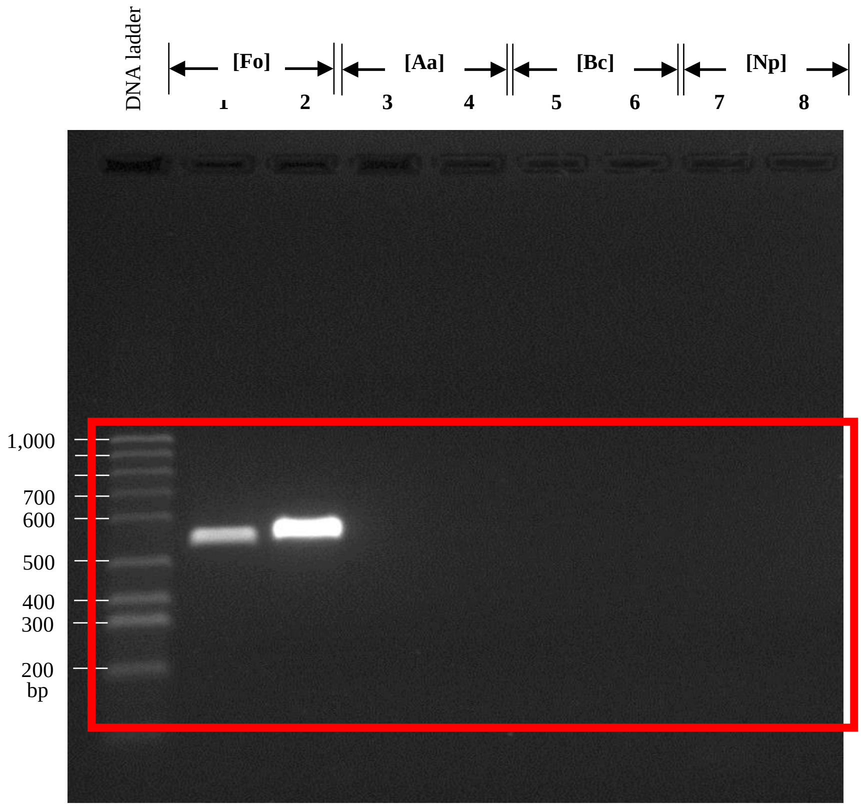

Generated image

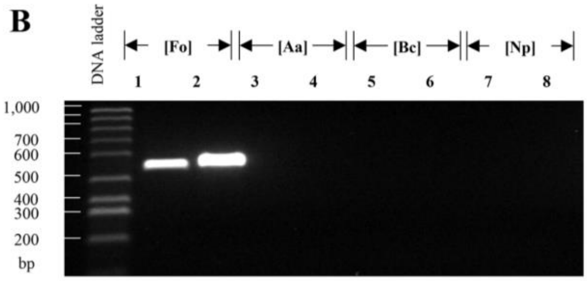

Fig. 10 (Figure legend).

PCR analysis of the PC03 marker previously reported for the diagnosis of *F. oxysporum*. From left to right are shown the PCR products obtained for *Fusarium kuroshium* [Fk], *F. graminearum* [Fg], *F. verticillioides* [Fv], *F. oxysporum* [Fo], *F. tricinctum* [Ft], *F. solani* [Fs], *A. alternata* [Aa], *B. cinereal* [Bc], and *N.parvum* [Np], lanes 1 to 9, respectively.

Notes.
The photographs were taken with Gel DocTM XR+ Imaging system, using Image Lab softwareTM (BIORAD). The red line box represents the gel area used to generate the figure. Brightness and contrast were slightly-adjusted on the entire image in order to improve visual aspect, is clear that this process NO misrepresent any results or information discussed in the main text.

Original image

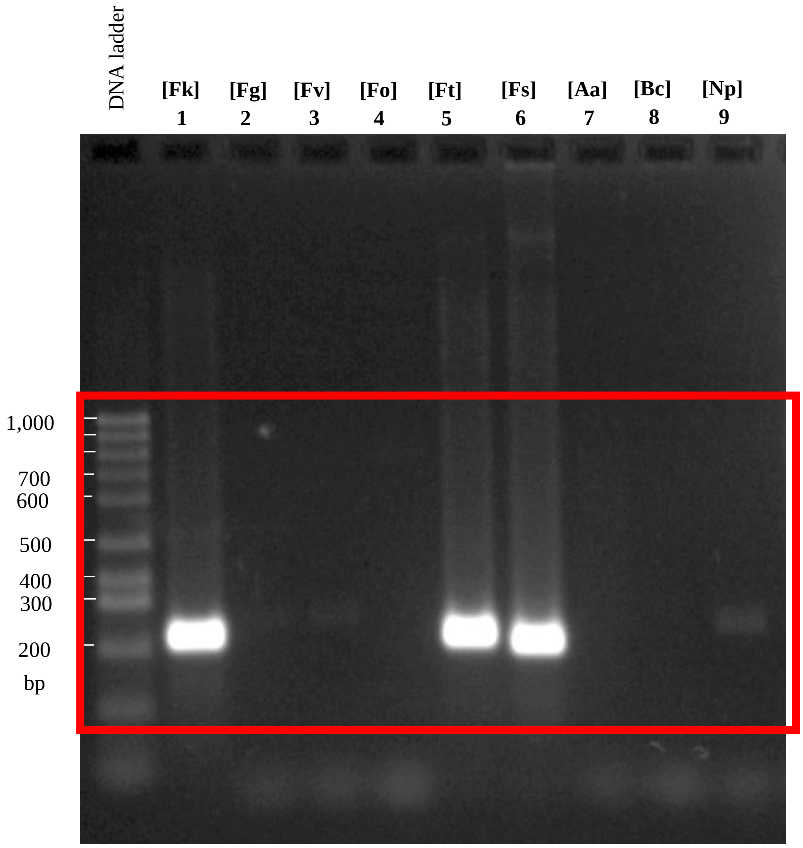

Generated image

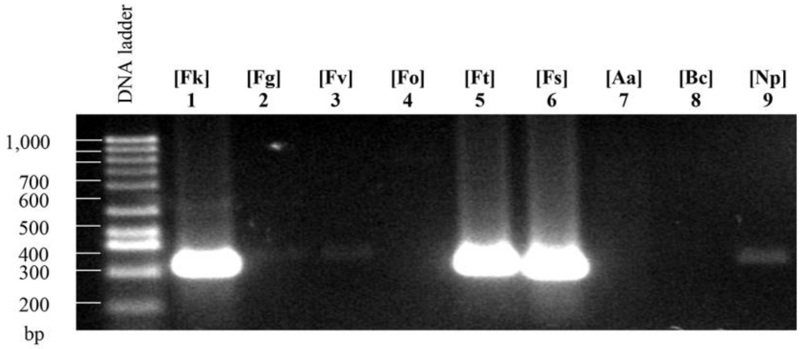

Supplement: S1 Raw images — (PDF) [file pone.0246079.s006.pdf]
